# A neutrosophic clustering approach to handle recommendation uncertainty for gray sheep users

**DOI:** 10.1038/s41598-026-41651-8

**Published:** 2026-03-21

**Authors:** Dina Samir, Eman Abd El Reheem, Saad M. Darwish, Magda M. Madbouly

**Affiliations:** 1 Department of Information Technology, Institute of Graduate Studies and Research, Alexandria University, Alexandria, Egypt; 2 Faculty of Computer and Data Science, Alexandria University, Alexandria, Egypt

**Keywords:** Gray Sheep, Neutrosophic Clustering, Collaborative Filtering, Recommender Systems, Engineering, Mathematics and computing

## Abstract

Recommender systems play a crucial role in reducing information overload by providing personalized suggestions based on user preferences. However, gray sheep users are individuals whose preferences are inconsistent or partially aligned with both majority and minority groups, overlooked by conventional collaborative filtering methods, resulting in inaccurate or inconsistent recommendations. To address this challenge, this paper introduces an uncertainty-aware integration framework for gray sheep users, combining neutrosophic k-means clustering with item-based collaborative filtering (IBCF). Neutrosophic logic is an extension of fuzzy logic that describes data using three membership degrees: truth (support), indeterminacy (uncertainty), and falsity (contradiction), allowing for the flexible and reliable identification of users with ambiguous or non-conforming behaviors. IBCF uses item similarity to generate customized recommendations in the high-indeterminacy (gray sheep) cluster, whereas standard item-based collaborative filtering is used to process mainstream users. The performance gains for the high-indeterminacy (gray sheep) cluster are the only focus of this study. Experimental evaluation on the MovieLens 100 K dataset demonstrates that the proposed model improves gray sheep user treatment significantly, achieving higher precision (88.70%), recall (90.90%), F1-score (89.79%), reduced error rates (MAE = 0.534, RMSE = 0.719) and accuracy (84.07%) presented as an additional indication. In addition, neutrosophic k-means is evaluated on Book-Crossing and Last.fm, demonstrating generalizability beyond movies. These results confirm that explicit uncertainty modeling within collaborative filtering architectures can improve the quality of identification and recommendations for gray sheep users, reaching out to a key segment of the user base that conventional techniques have neglected.

## Introduction

Based on user choices, recommender systems (RS), a type of information technology created to tackle information overload, generate personalized recommendations for products such as stocks, films, or book^1,2^. These systems aim to enhance the quality of information access services by reducing the time users spend searching for information while introducing them to resources they might not have otherwise discovered^3^. Numerous ethical and technical issues still plague recommendation systems, restricting their widespread use and efficacy. The cold start problem, in which systems lack sufficient information to accurately predict new users or products, is a recurring issue. This becomes more challenging with issues related to scalability and data sparsity, particularly in large-scale settings involving millions of individuals and items. Complexity is increased by integrating multi-modal data, such as text reviews, photos, and audio, necessitating sophisticated algorithms that can extract and combine heterogeneous data. Furthermore, since recommendation algorithms can unintentionally reinforce social injustices or compromise user privacy, ethical issues, including bias, fairness, and privacy, are becoming increasingly important^4^.

RSs employ three different methods to determine which items to recommend: content-based filtering (CBF), collaborative filtering (CF), and hybrid approaches that combine the two^5^. CF systems make recommendations for users based on the interests and preferences of others. These systems fall into two categories: model-based (MB) and memory-based (MRB). The MRB method collects data for the target user and makes recommendations based on similarities between users or items^6^. Both item-based and user-based approaches are included. The MB method, on the other hand, creates a model to forecast user preferences or ratings for specific items, and then uses those predictions to provide recommendations^7^. CF faces many difficulties, such as the gray sheep problem, which occurs when users with particular preferences receive less accurate recommendations, sparsity because of limited user ratings, the cold start problem, which is brought on by insufficient data for new users or items, and scalability issues as the number of users and items increases^8^.

CBF, on the other hand, makes recommendations for items that are comparable to those the customer has already liked based on user profiles and item descriptions. It suggests novel items that fit the user’s interests by matching user attributes like age, gender, location, and rated items with items of similar features^9^. Although these systems mostly rely on an extensive assessment of item attributes, they improve privacy by not requiring personal data. Nevertheless, they do not include user reviews, which restricts their capacity to evaluate product quality directly in the recommendations^10^. To build on their advantages, improve performance, and tackle issues like over-specialization, hybrid recommendation systems combine collaborative and content-based filtering techniques. Even if these systems broaden the range of recommendations, they still have problems with sparsity, early rater difficulties, and the cold-start problem^10,11^. Because gray sheep users have unusual or distinctive tastes, it can be difficult to find common ground with other system users^12^. Gray sheep consumers in e-commerce recommender systems have a big impact on how well- focused marketing works. Businesses must identify their clients and the products that best suit their interests to deliver customized ads or exclusive deals. As a result, the main difficulty is putting a gray sheep user into a certain user profile subgroup. To reflect various application situations and assess cross-domain resilience, we evaluate the proposed neutrosophic k-means clustering-IBCF framework on a variety of benchmarks, including MovieLens, Book-Crossing (books), and Last.fm (music).

The concept of gray sheep users in recommender systems is closely linked to the uncertainty in generating trustworthy recommendations. Because gray sheep users have unique or unusual interests that do not align with the bulk of users, collaborative filtering algorithms have a hard time predicting their preferences. Uncertainty has increased since gray sheep users have low correlation coefficients with other users, making it difficult to find comparable users for reliable recommendations^13^. Furthermore, this problem is made worse by data sparsity, which makes it more difficult to produce correct predictions due to insufficient data points^13^. When dealing with gray sheep users, collaborative filtering algorithms that depend on user-to-user similarities fall short, producing recommendations that are less trustworthy and have higher error rates. Some strategies to reduce this uncertainty include using clustering algorithms to find gray sheep users and better customize recommendations^14^. Another strategy is to use hybrid models that combine content-based filtering and collaborative filtering to use more user preference data^15^. By addressing the uncertainty that gray sheep users provide, recommender systems can perform better overall and produce recommendations that are more accurate and satisfying. To address inconsistent, ambiguous, and insufficient information, Smarandache developed neutrosophic sets^16^. By describing data points as a triple $\:\left(T\left(x\right),I\left(x\right),F\left(x\right)\right)$, where $\:T\left(x\right)$ stands for truth, $\:I\left(x\right)$ for indeterminacy, and $\:F\left(x\right)$ for falsity, the neutrosophic logic is based on fuzzy logic. By combining degrees of membership, non-membership, and indeterminacy, the neutrosophic k-means clustering algorithm used in this study is very successful in handling ambiguous and incomplete data^17^. Because it can capture the ambiguity and unique preferences of users whose behavior differs from others,’ this method helps detect gray sheep users. The high dimensionality in collaborative filtering and the sparsity of data makes it difficult for modern recommender systems to handle unusual user behavior, particularly from gray-sheep users.

In memory- and model-based collaborative filtering, each user is represented in a space whose dimensionality equals the number of available items, and each item in a space whose dimensionality equals the number of users, resulting in user-item matrices with thousands of dimensions, even for medium-sized datasets like MovieLens 100 K and 1 M. The high-dimensional rating space leads to unstable similarity estimates and exacerbates the gray-sheep problem, as users with abnormal patterns get lost in a noisy, sparse feature space. Sparsity refers to the fact that only a small fraction of the possible user-item pairs are actually rated, so the majority of the matrix entries are missing. For example, the 100 K ratings over 943 users and 1,682 movies in MovieLens 100 K correspond to a density of only about 6.3%, implying that nearly 94% of potential ratings are missing. In sparse, high-dimensional situations, standard CF fails to accurately describe users whose behavior differs from the majority, particularly gray-sheep users. As a result, gray sheep users are embedded in a highly sparse, high-dimensional rating space where measurements based on correlation and distance become inaccurate for building neighborhoods. To tackle this, a number of techniques have been developed, including deep learning, social network analysis, distribution-based detection, clustering, and one-class classification. Although these approaches provide trade-offs between accuracy, scalability, and complexity, there are still issues with interpretability, robust user modeling, and integrating auxiliary data into practical settings^18^.

By learning decision boundaries in the user-item rating space, one-class classification and outlier detection frameworks produce prediction models that differentiate between normative and gray sheep behaviors^19,20^. To find users with unusual preference patterns, these techniques use a range of algorithms, such as principal component analysis, one-class support vector machines, k-nearest neighbors (KNN), local outlier factor, and autoencoder variations. Since misclassifying mainstream users as anomalies can impair overall system performance and user satisfaction, two major challenges are creating robust user representations in extremely sparse, high-dimensional spaces and adjusting detection thresholds to balance false positives and false negatives^20^. Using hybrid text classification techniques for profile enrichment or modified k-means algorithms (e.g., k-means + + and power-weighted variants), clustering-based approaches identify gray-sheep users offline by grouping users whose similarity to all other clusters falls below an empirically determined threshold^14^. Following isolation, specialized recommenders produce recommendations specific to these clusters, such as switching hybrids or pure content-based filters. These approaches, however, struggle to choose the ideal number of clusters, adapt to changing user preferences over time, and retain computing efficiency when scaling to millions of users and items.

Distribution-based identification methods, which employ ideas from information retrieval and density estimation to find outliers without the need for explicit clustering or network data, identify gray sheep users by identifying abnormalities in the statistical distribution of paired user-user similarity values. To prevent over- or under-detection of anomalous users, the fundamental problem is to accurately describe similarity distributions in the context of noisy, sparse ratings and to define principled criteria that generalize across diverse datasets and domains^1^. By establishing hybrid similarity metrics that include rating concordance and homophily effects, social network community detection techniques take advantage of associations inferred from explicit social ties or implicit co-interaction graphs to enhance suggestions for users with niche interests. These methods exploit users’ network neighborhoods to help minimize sparsity. However, they must deal with the difficulty of integrating multi-modal data sources in real time and need access to trustworthy social or trust data, which is frequently unavailable because of privacy restrictions^13^.

To densify the neighborhood structure and increase prediction accuracy in sparse regions, deep learning and virtual user inference techniques employ neural architectures, particularly autoencoders, to supplement the rating matrix with synthetic users whose preferences are inferred as opposites of real users. By modifying latent representations learned from real users, often with the aid of autoencoders, virtual user inference techniques create synthetic user profiles. By simulating believable but opposing tastes, these synthetic users improve collaborative filtering and help close data gaps. Although these methods have shown improvements in root mean square error (RMSE) and mean absolute error (MAE). On benchmarks like MovieLens 100 K, they have drawbacks, including high training costs, interpretability issues (black-box nature), and the possibility of overfitting to reconstruction noise in the rating data^21^. To create an enhanced rating matrix that contains both actual and inferred virtual user evaluations, matrix-factorization with association-rule enrichment techniques combines association-rule mining with singular value decomposition or other latent- factor models. These hybrid approaches solve the sparsity and gray-sheep problems while increasing neighborhood density by including auxiliary relations, such as item co-occurrence patterns. The primary disadvantages are the necessity to balance the impact of association-derived items against real user feedback, sensitivity to the ratio of actual to virtual data, and the increased computing complexity of updating and maintaining enriched matrices^22^.

The recommendation system described in this study addresses the gray sheep problem in collaborative filtering using a distinct suggestion strategy: Users are segmented using neutrosophic k-means clustering based on uncertainty patterns, with customized item-based collaborative filtering (IBCF) applied to the high-indeterminacy (gray sheep) cluster. Neutrosophic clustering works as an uncertainty detection layer, identifying individuals with various and ambiguous preferences by assigning neutrosophic degrees of truth, indeterminacy, and falsity. Users in the high-indeterminacy cluster receive recommendations via IBCF, which uses item similarity to make personalized suggestions. Standard item-based collaborative filtering is used for mainstream users, establishing a baseline setting for the study. The main contribution is an improved identification and recommendation pipeline for the gray sheep cluster. An experimental comparison shows that the proposed framework improves existing clustering-based standards, with a focus on the gray sheep section. Overall, the work contributes to the system-level integration of proven methodologies inside an uncertainty-aware framework aimed at better managing gray sheep users. This paper’s remaining sections are organized as follows: Section 2 describes the related literature of recommendation systems. Section 3 has model discussion. Section 4 illustrates the experimental results. The conclusion and future work are presented in Section 5.

## Related work

Numerous studies have focused on improving RSs by addressing persistent issues, including data sparsity, cold-start challenge, and concerns related to gray sheep. This section provides a critical evaluation of several pertinent studies, highlighting the proposed methodological advancements and their contributions to the field’s progress. “Table 1” outlines the strategies used in these typical investigations, as well as the major issues addressed (such as sparsity, cold start, and gray sheep behavior), attained performance, and remaining limits.

**Table 1:** Summary of representative related work on recommender systems, highlighting techniques, addressed challenges, and remaining limitations in handling data sparsity, cold-start problems, and gray sheep user behavior.

Ref.	Authors	Technique/method	Main problems addressed	Performance metrics	Key limitations
^19^	Alabdulrahman & Viktor	One-class ML approaches for gray sheep detection	Model gray sheep users whose tastes deviate from mainstream/niche	F1-score = 0.81	Risk of overfitting on skewed data; limited interpretability
^23^	Cai et al.	Many-objective evolutionary algorithm hybrid (CBF + CF)	Joint optimization of accuracy, diversity, novelty, coverage	NDCG = 0.79 (MovieLens)	High computational cost; poor scalability for large, real-time systems
^24^	Monsalve-Pulido et al.	Autonomous hybrid recommender for virtual learning	Real-time adaptation to heterogeneous learner needs	Precision = 0.85	Complex real-time processing; latency on large datasets
^25^	Kulkarni & Rodd	Context-aware recommendation (time, location, preferences)	Higher personalization via contextual factors	Improved precision, recall, F1-score vs. non-contextual	Adds complexity; aggravates sparsity; real-time processing unresolved
^26^	Furtado & Singh	ML-driven recommender (DT, KNN, SVM with feature engineering)	Capture complex user-item interactions	82% accuracy	Requires large training data; heavy tuning; cold-start vulnerable
^27^	Zhang et al.	Online learning + context-aware engine (CF with temporal signals)	Real-time updates as preferences evolve	Precision = 0.82, Recall = 0.76	High resource demands; complex low-latency at scale
^28^	Zhou	Deep learning (embeddings) for e-commerce	Model rich, nonlinear user-item interactions	AUC = 0.91	High computational complexity; poor real-time suitability
^29^	Chen et al.	Knowledge-graph-based conversational recommender (DL + RL)	Interactive, multi-turn recommendations	Task completion = 72.4%, BLEU = 0.195	Depends on KG quality; high complexity; limited cross-domain use
^30^	Selvi & Sivasankar	Modified fuzzy c-means with partial membership degrees	Capture ambiguous overlapping preferences	MAE = 0.68, RMSE = 0.85	Sensitive to initialization and cluster count
^31^	Chen, Lin, & Thaipisutikul	CF with dynamic time decay mechanism	Emphasize recent interactions	RMSE = 0.861, MAE = 0.682	Extra computational burden; degradation at scale
^32^	Wang et al.	Hybrid CF (user-based + item-based) for medical services	Handle healthcare sparsity and sensitivity	Precision = 0.81, Recall = 0.78	Cold-start unresolved for new users/services
^33^	Mishra & Rathi	Scalable job recommender (CF + matrix factorization)	Minimize overhead on large platforms	Precision = 0.83, Recall = 0.79	Persistent cold-start for new users
^34^	Lv et al.	Hybrid CF + knowledge-graph reasoning for e-commerce	Reduce sparsity and cold-start via semantics	Precision = 0.84, Recall = 0.81	KG construction/maintenance complex and resource-intensive
^35^	Ifada, Putri, & Sophan	Normalization-based multi-criteria CF	Standardize multiple rating dimensions	MAE = 0.72	Requires multi-criteria ratings unavailable in standard datasets
^36^	RaviKanth et al.	Hybrid: MF (model-based) + similarity (memory-based)	Combat sparsity and overfitting	Accuracy = 0.85, Recall = 0.82	Added complexity; increased dual pipeline computation
^37^	Ma et al.	Multi-objective optimization (diversity + accuracy)	Diverse recommendations + relevance	F1-score = 0.84; +17.5% diversity	High complexity; becomes unaffordable at scale
^38^	Suganeshwari & Syed Ibrahim	Rule-based CF using explicitly disliked items	Negative feedback to avoid unwanted items	Precision = 0.89, Recall = 0.86	Explicit negative feedback sparse in practice
^39^	Zhang et al. (UR)	User-based CF with trust mechanism + time weighting	Adapt to changing preferences; reliability	Precision = 0.84, Recall = 0.81	Trust/time computation overhead; scalability concerns
^40^	Sánchez-Moreno et al.	Session-based song recommendation	Personalization in short sessions; cold-start	Precision = 0.82, Recall = 0.78	Sensitive to session variability; limited beyond music
^41^	Srivastava, Bala, & Kumar	Hybrid models with user-centric metrics for gray sheep	Better classify gray sheep; reduce irregular impact	+ 12% precision; improved accuracy/diversity	Scalability challenges with diverse behavior

Using a many-objective evolutionary algorithm, Cai et al.^23^ presented a hybrid recommender that combines content-based techniques with collaborative filtering to optimize accuracy, diversity, novelty, and coverage jointly. As demonstrated by a normalized discounted cumulative gain of 0.79 on MovieLens, this produces more user- focused, balanced recommendations while improving diversity and novelty without sacrificing accuracy. Many-objective optimization’s primary disadvantage is its high computing cost, which could make it difficult for large, real-time systems to scale. By combining content-based and collaborative filtering methods, Monsalve-Pulido et al.^24^ created an autonomous hybrid recommender for virtual learning that achieves a precision of 0.85 through real-time adaptation and a feedback loop that tailors resources to different learner needs. Its flexibility and ongoing accuracy improvement are its key advantages, but when expanding to big datasets, its complex nature in real-time data processing may cause latency. To provide more relevant recommendations, Kulkarni and Rodd^25^ provided a thorough analysis of context-aware recommendation systems that consider situational elements like time, location, and user preferences. Compared to conventional approaches, the benefits include highly customized recommendations that adjust to users’ shifting contexts, increasing relevance and enjoyment. Modeling a variety of contextual variables adds complexity, which can increase processing costs and make data sparsity problems worse. Context-aware techniques routinely surpass non-contextual baselines in accuracy and user satisfaction, according to objective criteria like precision, recall, and f1-score. However, real-time processing and handling vast amounts of contextual input continue to be unresolved issues. To better capture complex user–item interactions, Furtado and Singh^26^ presented a machine learning-driven movie recommender that combines feature engineering in explicit and implicit user feedback with decision trees, KNN, and support vector machines (SVM). Their method’s benefits include far better recommendation relevancy thanks to advanced algorithms and characteristics that capture complex preference patterns. Some drawbacks include the significant computational resources and parameter tuning needed for scalability, as well as its strong reliance on sizable training datasets, which leaves it open to cold-start situations with sparse data or new users. The accuracy of 82% in forecasting user preferences provides an objective measure of its efficacy, demonstrating both high performance and the necessity of careful data management and system optimization in large-scale deployments.

Using online learning and a context-aware engine, Zhang et al.^27^ created a personalized, real-time movie recommender that combines collaborative filtering with contextual and temporal signals to instantly modify recommendations as user preferences and behavior change. Among its benefits are its real-time flexibility, which keeps recommendations current and extremely relevant, increasing user pleasure and engagement. The main drawbacks are the resource requirements and engineering complexity needed to maintain low-latency performance at scale, particularly when handling high volumes of contextual data and interactions. Strong accuracy and responsiveness are demonstrated by objective measurements in empirical tests, which also illustrate the trade-off between system complexity and live adaptability. The precision and recall are 0.82 and 0.76, respectively. Zhou^28^ presented a deep learning-based e-commerce product advertising recommender that models rich, nonlinear user-item interactions using distributed representations, or embeddings. The benefit of this approach is that, even in high-dimensional, sparse contexts, it may reveal intricate patterns in behavior and product features, producing recommendations that are incredibly precise and tailored. Adoption by smaller platforms or in low-latency, real-time environments may be hampered by its significant computing complexity and reliance on large-scale training data. An area under the curve (AUC) of 0.91 provides objective evidence of its predictive power, indicating significant gains over conventional recommendation methods while also highlighting the necessity for additional optimization to balance model complexity with real-time demands.

A knowledge-based dialogue recommender that combines deep learning and reinforcement learning with knowledge graphs to generate interactive, multi-turn recommendations that are suited to changing user intents was presented by Chen et al.^29^. Benefits include its ability to use structured knowledge to make more contextually relevant and comprehensible recommendations, as well as its ability to change dynamically through reinforcement learning and graph-based reasoning. The increased system complexity and heavy dependence on the completeness and quality of the underlying knowledge graph are drawbacks that may limit scalability and cross-domain generalizability. Its ability to generate coherent, purposeful dialog-based recommendations is demonstrated by objective measurements of its performance, specifically a task- completion success rate of 72.4% and a bilingual evaluation understudy (BLEU) score of 0.195 for language coherence. However, it also highlights areas for improvement in conversational fluency and robustness. In order to better capture ambiguity and overlapping user preferences, Selvi and Sivasankar^30^ presented a recommender optimization based on a modified fuzzy c-means clustering that awards partial membership degrees. Because it can model subtle preference overlaps, this soft-clustering strategy has the advantage of better clustering accuracy. It also has the advantage of faster, more robust convergence through an improved similarity measure. Their sensitivity to initialization and the number of clusters selected are drawbacks, as improper tuning can have a major effect on performance. When compared to traditional clustering- based recommendation approaches, their tests’ objective measurements reveal an MAE of 0.68 and an RMSE of 0.85, indicating greater prediction accuracy.

A collaborative filtering system enhanced with a dynamic time decay mechanism that down-weights past user–item interactions in favor of more current behavior was introduced by Chen, Lin, and Thaipisutikul^31^. Because the decay function makes sure that new user preferences have a bigger impact on the suggestion process, this design has the advantage of being more accurate and timely. The additional computational burden of calculating and applying the time decay has drawbacks, which, if not executed well, might impair performance in large-scale installations. The model’s RMSE of 0.861 and MAE of 0.682, as determined by objective measurements from benchmark studies, demonstrate significant accuracy improvements over static-weight baselines and highlight the importance of temporal dynamics in collaborative filtering. A collaborative filtering-based recommender designed for online medical services was created by Wang et al.^32^, who used patient history and preference data to pro- vide pertinent healthcare recommendations. Benefits include its careful handling of healthcare-specific issues like data sparsity and sensitivity, which eventually improve patient accessibility and decision-making, and its dual use of user-based and item- based collaborative filtering, which improves recommendation accuracy by capturing both similar patients’ behaviors and analogous services. The cold-start problem has drawbacks: early recommendations are less trustworthy when new users or innovative services do not engage with data enough. Despite the limitations of real-world data, objective measurements in their trials show a precision of 0.81 and a recall of 0.78, indicating the system’s good performance in suggesting appropriate medical treatments.

In order to minimize computational overhead and preserve high-quality recommendations on extensive employment platforms, Mishra and Rathi^33^ introduced a scalable job recommender that optimizes user-item similarity calculations through the use of collaborative filtering and matrix factorization. Their method’s benefits include its capacity to analyze large datasets quickly and effectively for responsive matching, as well as its integration of implicit feedback signals like clicks and views that enhance personalization beyond explicit evaluations. The main drawback is the ongoing cold-start issue, which means that until enough data is gathered, new users with less interaction history can get fewer accurate recommendations. A precision of 0.83 and a recall of 0.79 in real-world assessments provide objective evidence of the system’s efficacy, demonstrating its robust performance in dynamic, high-volume job suggestion circumstances. In order to improve the accuracy and semantic relevance of recommendations in e-commerce contexts, Lv et al.^34^ introduced a hybrid e-product recommendation system that incorporates collaborative filtering with knowledge graph reasoning. This method’s benefits include its ability to reduce sparsity and cold-start issues by utilizing semantic linkages and its utilization of structured product features and relationships to provide more intelligent, explicable recommendations. The knowledge graph’s increased complexity and maintenance, which require a large amount of computer power and subject knowledge, are drawbacks. Their studies’ objective measurements show a precision of 0.84 and a recall of 0.81, which outperforms conventional collaborative filtering techniques.

Ifada, Putri, and Sophan^35^ introduced a normalization-based multi-criteria collaborative filtering strategy that considers many user rating dimensions, including quality, usability, and satisfaction, to increase the accuracy and fairness of recommendation systems. The ability to standardize and integrate many evaluation criteria yields more thorough, user-aligned suggestions that lessen bias and improve forecast consistency, among other benefits. The additional data needed for multi-criteria ratings are a drawback, as they cannot be easily accessible in typical datasets, which restricts their use. When the system’s performance is objectively measured, MAE is 0.72, indicating that it is more accurate than conventional single- criterion collaborative filtering techniques. RaviKanth et al.^36^ presented a hybrid e-commerce recommender that combines model-based approaches (matrix factorization) with memory-based approaches (user-user and item-item similarity) to use the predictive power and scalability of the latter and the clarity of the former. By utilizing complementary capabilities, this dual method has the advantage of being able to deal with overfitting and data sparsity. While model-based filtering provides strong generalization on big datasets, memory-based filtering provides obvious insights into similarity. The additional system complexity and computing burden needed to integrate, improve, and maintain two different algorithms simultaneously are a major drawback. The system demonstrated its efficacy in generating precise and scalable suggestions for dynamic online shopping environments by achieving an accuracy of 0.85 and a recall of 0.82 in empirical testing conducted on an e-commerce dataset.

A multi-objective optimization-based recommender that manages variety and accuracy as Ma et al.^37^ proposed simultaneous goals. This framework’s ability to provide a greater variety of item recommendations while retaining a high level of relevance to user preferences increases exposure to new material. The substantial processing complexity needed to solve multi-objective problems, which can become unaffordable as user and item counts increase, is a drawback. The system’s ability to provide well-rounded recommendations that encourage long-term involvement is demonstrated by objective metrics from their studies, which show an F1-score of 0.84 and a 17.5% improvement in a diversity index over baseline models.

To improve personalization, Suganeshwari and Syed Ibrahim^38^ presented a rule- based collaborative recommendation system that specially uses expressly disliked items. Their method’s benefits include the incorporation of negative feedback, which stops the system from recommending things that users do not want, increasing user happiness and trust. Another benefit is the openness and explainability that come with a rule-based system. When consumers infrequently indicate items they dislike, the method’s usefulness may be limited due to the possible sparsity of explicit negative feedback in real-world datasets. A precision of 0.89 and a recall of 0.86 on benchmark datasets provide objective evidence of the system’s efficacy, surpassing conventional collaborative filtering methods and highlighting the importance of considering both positive and negative user comments. In order to adapt to changing preferences and weed out outdated or unreliable data, Zhang et al.^39^ presented UR, a user-based collaborative filtering recommender that combines a trust mechanism, evaluating user reliability via rating behavior, with time weighting to emphasize recent interactions. UR’s benefits include improved recommendations’ relevance and trustworthiness through temporal focus and trust assessment. Achieving a precision of 0.84 and a recall of 0.81, which surpasses conventional collaborative filtering baselines, shows that it is effective, but it has drawbacks, including the additional computational load of calculating trust scores and updating time-based weights, which may hinder scalability. The advantages of combining temporal dynamics with trustworthiness for more accurate, context-aware recommendations are demonstrated by this work.

Sánchez-Moreno et al.^40^ suggested a session-based song recommendation system that improves personalization in brief listening sessions by using user characterization based on the play power-law distribution. Without relying on long-term history, the approach dynamically simulates user behavior, which makes it especially appropriate for cold-start or temporary scenarios. Its sensitivity to session variability and limited ability to be generalized outside of music streaming may be its drawbacks, despite its superior contextual responsiveness. The system’s ability to provide tailored recommendations based on short-live behavioral patterns was validated by its evaluation, which showed a precision of 0.82 and a recall of 0.78. Using one-class machine learning approaches, Alabdulrahman and Viktor^19^ presented a recommendation approach tailored for gray sheep users, whose tastes deviate from mainstream and specialized patterns. Their method addresses data sparsity and enhances personalization for these unusual users by modeling user behavior based only on positive input. The skewed nature of one-class data may cause overfitting, even while the strategy improves diversity and relevance in suggestions. With an F1-score of 0.81, the method proved successful in customizing recommendations to suit individuals with non-conforming tastes.

Srivastava, Bala, and Kumar^41^ investigated gray sheep user behavior in e-commerce, who looked at how their irregular preferences impact the performance of the recommendation. In order to better classify and assist these individuals, the study developed user-centric metrics and hybrid models that combine contextual and behavioral data. The complex nature of modeling such diverse behavior poses scalability issues, even though the suggested techniques increased precision by up to 12% and improved both accuracy and diversity of recommendations. The necessity of inclusive and flexible recommendation systems for users with non-conforming rating patterns is emphasized by this work. Because of its exceptional capacity to manage uncertainty and unusual user behavior, especially that of gray sheep users, while preserving scalability and personalization, neutrosophic clustering combined with IBCF stands out as a superior strategy when compared to current recommender system approaches. In order to empirically show that a neutrosophic k-means clustering-IBCF pipeline may better serve gray-sheep users by explicitly capturing indeterminacy, we incorporate NCF, LightGCN, and AutoRec implementations as modern neural baselines in our experiments.

Deep learning for cross-domain sequential recommendation (CDSR) now incorporates semantic enrichment, relational reasoning, and multimodal fusion to accurately represent user preferences. Wu et al.^42^ introduce Tag-Enriched Multi-Attention with Large Language Models (TEMA-LLM), which uses LLMs to generate semantic tags from item titles/descriptions and fuses them with item, text, and visual embeddings via a multi-attention mechanism to jointly model intra- and inter-domain user preferences, outperforming baselines on large e-commerce datasets due to enriched semantics. However, it still heavily depends on textual tag quality and may underutilize visual data in sparse settings (TEMA‑LLM). Relation-Fused Attention in Knowledge Graphs (RFA-KG) improves latent semantics beyond interaction histories and reduces sparsity by incorporating knowledge graph relational information through cross-compression and dynamic gating. However, it does not explicitly model multimodal item content or sequential behavior in CDSR contexts^43^. Although the computational cost of LLM inference is still a problem, LLM-Enhanced Multimodal Fusion (LLM-EMF) shows superior performance in multimodal integration by augmenting textual and visual item representations with LLM knowledge and multiple attention layers to capture both single-domain and cross-domain preferences^44^. Image Fusion for Cross-Domain Sequential Recommendation (IFCDSR) improves visual preference capture by using multi-attention to jointly learn intra- and inter-domain preferences and incorporates CLIP-based visual embeddings to enhance item representations. However, it lacks deeper relational reasoning and explicit semantic tagging^45^. Uncertainty is implicitly absorbed into dense latent representations and loss functions in these CDSR models rather than explicitly quantified, so high-indeterminacy gray-sheep users are treated as noise in the optimization process rather than as a distinct segment requiring specialized handling. Our neutrosophic k-means clustering-IBCF framework focuses on uncertainty-aware gray-sheep user segmentation within a single-domain collaborative filtering setting, in contrast to these deep learning-based CDSR architectures that aim for cross-domain sequential representation learning with rich multimodal and LLM-enriched content. Instead of relying exclusively on point estimates from deep models that minimize global reconstruction or ranking error, the suggested approach explicitly incorporates truth, indeterminacy, and falsity degrees, transforming uncertainty into a useful supervisory signal for detecting and supporting gray-sheep users. It can be integrated as a complementary gray-sheep handling component rather than a cross-domain sequential backbone.

Neutrosophic clustering naturally models indeterminacy, which enables it to identify users with inconsistent or non-conforming preferences without requiring a lot of con- textual input, unlike computationally demanding many-objective algorithms^23^ or context-aware systems^25,27^ that call for complicated modeling and extensive con-textual data processing. Due to their strict assumptions or reliance on sizable, labeled datasets, traditional clustering (such as fuzzy c-means^30^ or machine learning-based models^26,28^ may miss hidden user patterns. This offers a simple yet efficient method for doing so. Additionally, neutrosophic clustering permits partial and uncertain membership, better-capturing preference ambiguity and enhancing generalizability than one-class classifiers created for gray sheep users^19^, which run the danger of over-fitting due to skewed feedback. This hybrid approach provides robust, scalable, and personalized results when paired with IBCF, which is excellent at using item similarity to generate accurate and interpretable recommendations. This is especially true in sparse or noisy environments where traditional systems, whether rule-based^38^, temporal^31^, or knowledge-graph-driven^29,34^, find it difficult to adapt. Thus, through a more balanced, scalable framework, neutrosophic clustering combined with IBCF enhances recommendation quality for underrepresented user groups. Therefore, our contribution lies in explicitly modeling indeterminacy to improve gray-sheep user handling inside a collaborative filtering pipeline, which is complementary to recent deep learning and cross-domain sequential recommendation models like TEMA-LLM, RFA-KG, LLM-EMF, and IFCDSR, which primarily advance representation learning across domains and modalities. In domains other than recommender systems, such as violence detection from cloud-based surveillance data, neutrosophic cognitive maps have been successfully applied to model complex uncertainty by explicitly encoding indeterminate factors to improve decision quality^46^. NCM-based neutrosophic analyses have also been used in educational settings to examine critical thinking through the TEC 21 model, demonstrating the applicability of neutrosophic logic for managing student data and uncertain expert evaluations^47^. Furthermore, Squacc BiLSTM combines deep learning with neural knowledge graphs for dense video captioning, illustrating how uncertainty-aware and semantically enriched representations can improve sequence understanding in multimedia tasks^48^.

## Methods

**Fig. 1:**
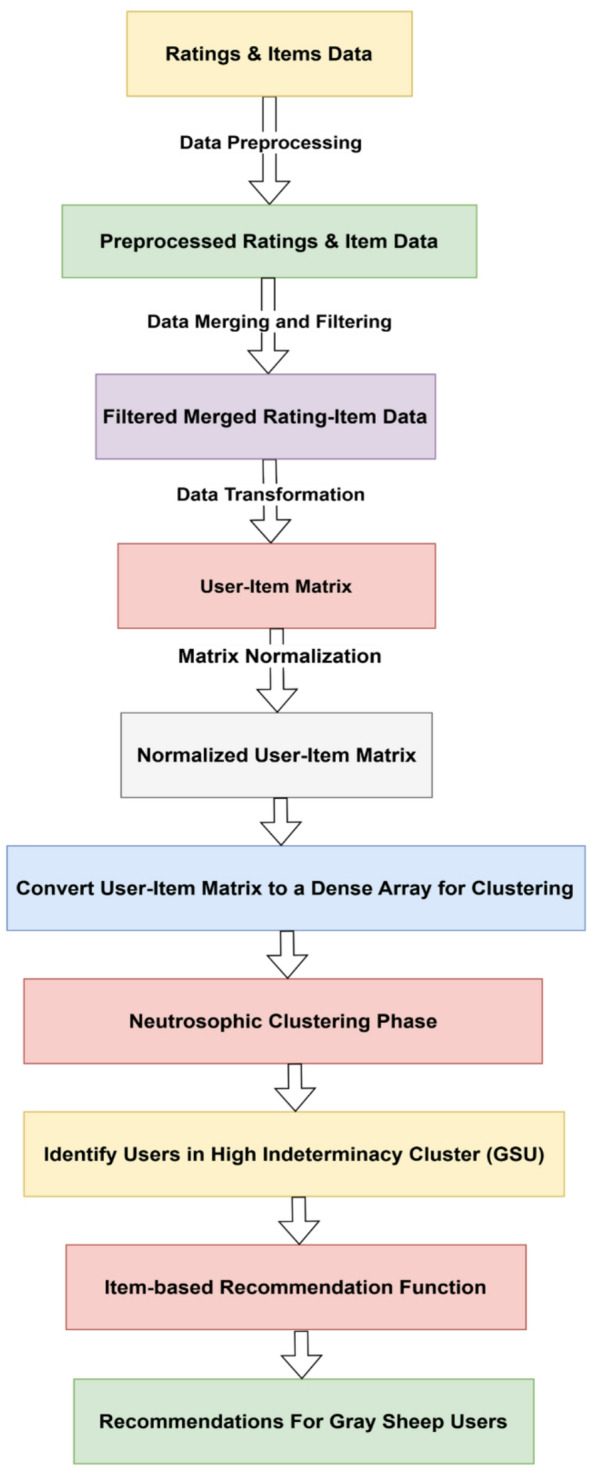
Neutrosophic K-means clustering recommendation model.

The proposed approach employs a user-segment-aware recommendation architecture that responds to various user behaviors via neutrosophic clustering. It divides people into mainstream and gray sheep categories, using regular IBCF for consistent users and an enhanced IBCF for those with ambiguous or inconsistent ratings. The primary goal is to improve recommendation accuracy for gray sheep users while maintaining consistent performance for others. “Figure 1” illustrates the suggested uncertainty-aware recommendation pipeline for gray sheep users. The method consists of four basic stages: (1) Data preprocessing and user-item rating matrix building, (2) neutrosophic k-means clustering to segment users based on uncertainty (indeterminacy), and (3) Item-based collaborative filtering (IBCF) for users in the high-indeterminacy (gray sheep) cluster.

### Data acquisition and preprocessing

#### Dataset

As mentioned in the official GroupLens documentation, the data source was the MovieLens 100 K dataset, which included 100,000 ratings from 943 people across 1,682 movies. The dataset is frequently supplemented with other metadata, such as movie names and genres, to provide more comprehensive analysis. Other experiments are carried out using Book-Crossing and Last.fm benchmarks to investigate generalizability in the book and music recommendation domains.

### Data preparation and matrix transformation

The MovieLens 100 K ratings and item metadata were integrated on movieId to create a user-item interaction matrix with 943 people and 1,682 movies, while respecting the data’s natural sparsity. Items with fewer than 100 ratings were deleted to ensure that similarity and clustering are based on well-supported interaction patterns. Ratings were mean-centered at the item level to decrease global popularity effects and stabilize Pearson correlation-based similarity assessments.

### Data normalization and similarity calculation

#### Normalization

To improve the accuracy of similarity estimates, the user-item matrix is represented in mean-centered form, with each rating stated in relation to the item’s average rating. This adjustment highlights relative user preferences and attenuates systematic biases caused by globally high- or low-rated items, which is usual practice in correlation-based collaborative filtering.

### Similarity score calculation

The Pearson correlation coefficient in “equation (1)” is used to calculate item-item similarity, which represents the linear link between the rating vectors of two items over users who have rated both. This measure naturally emphasizes overlapping ratings and implicitly corrects for user-specific rating scales, making it suitable for sparse collaborative filtering data. The similarity between items $\:i$ and $\:j$ is calculated using Pearson correlation, defined as^49^:

1 $$\:Sim\left(i,j\right)=\:\frac{{\sum\:}_{u\in\:Uij}\:\left({r}_{ui\:}-\:\:{r^{-}}_{i}\right)({r}_{uj\:}-\:\:{r^{-}}_{j})}{\:\sqrt{{\sum\:}_{u\in\:Uij}\:\:{\left({r}_{ui\:}-\:\:{r^{-}}_{i}\right)}^{2}}\:\:\:\:\:\:\sqrt{{\sum\:}_{u\in\:Uij}\:\:{\left({r}_{uj\:}-\:\:{r^{-}}_{j}\right)}^{2}}}\:$$

Where: $\:{U}_{ij}\:$denotes the set of users who have rated both items $\:i$ and $\:j$, $\:{r}_{ui\:}$and $\:{r}_{uj\:\:\:}$represent the ratings given by user $\:u$ for items $\:i$ and $\:j$, respectively and $\:{r^{-}}_{i}$ and $\:{r^{-}}_{j}$ are the average ratings of items $\:i$ and $\:j$.

### Sparse user-item matrix: gray sheep challenges and neutrosophic requirements

**Fig. 2:**
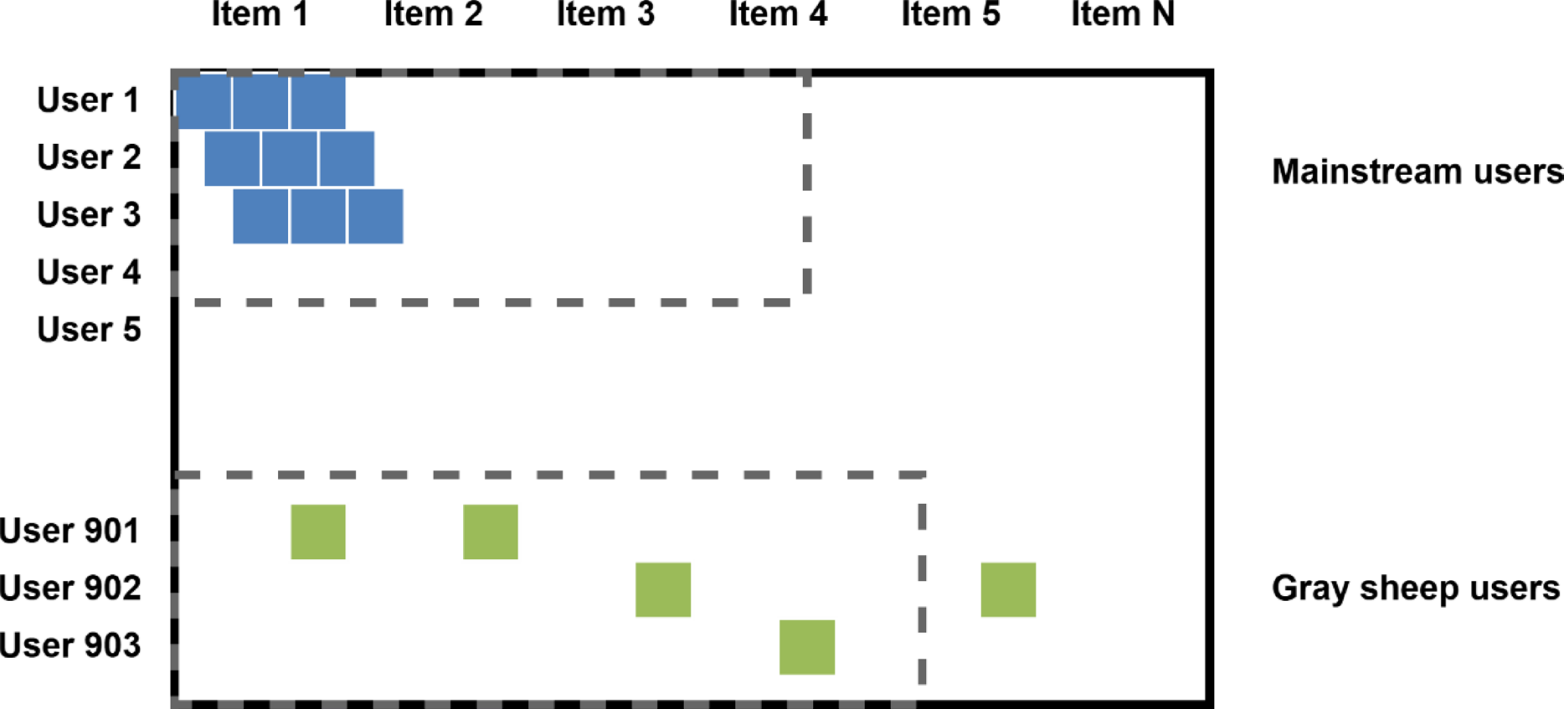
Sparse user–item rating matrix with mainstream and gray sheep users.

The user-item matrix is extremely sparse, with only around 6.3% of entries observed, making similarity estimation and grouping particularly difficult, especially for users exhibiting aberrant behavior^50^, as illustrated in “Figure 2”. In such matrices, mainstream users have coherent and consistent rating patterns, whereas gray sheep users have scattered and weakly connected ratings across objects. This finding highlights the need for a clustering method that can handle missing data without relying significantly on imputation, supports soft cluster membership, and clearly models indeterminacy for ambiguous users.

### Neutrosophic clustering for gray sheep detection

The neutrosophic k-means clustering approach improves on the classic k-means algorithm by including three membership components: truth ($\:T$), indeterminacy ($\:I$), and falsity ($\:F$), to effectively deal with incomplete and ambiguous data. This three-dimensional formulation allows each data point to describe its degree of cluster membership ($\:T$), assignment uncertainty ($\:I$), and non-membership ($\:F$) rather than being forced into a single crisp assignment. By modeling both characteristics concurrently, neutrosophic k-means may capture both unambiguous and uncertain cluster memberships, making it very useful for detecting people with unusual or inconsistent preferences, often referred to as gray sheep users.

### Matrix transformation for clustering

In order to get fixed-length user vectors appropriate for distance computation, missing ratings are only handled as zeros throughout the clustering stage. In order to prevent artificially inflating user-item interactions, the original sparse matrix is used for all recommendation evaluation.

### Cluster initialization and distance-to-similarity conversion

Cluster centroids are initialized by sampling user rating vectors, preventing bias towards any certain region of the feature space. The Euclidean distances between user profiles and centroids are then calculated^51^ as in “equation (2)”, and these distances are monotonically transferred to similarity scores, resulting in higher similarity for closer individuals. The resulting similarity values are fed into the neutrosophic membership functions, which connect geometric proximity in the rating space to the truth, indeterminacy, and falsity degrees of cluster membership.

2 $$\:distance=\left({X}_{j\:,\:}{C}_{i}\right)=\left|\left|{X}_{j\:\:-\:}{C}_{i}\right|\right|$$

### Neutrosophic membership degree calculation

The neutrosophic k-means model describes each user-cluster pair’s relationship using three components: truth $\:T\left(x\right)$, indeterminacy $\:I\left(x\right)$, and falsity $\:F\left(x\right)$, which measure membership, ambiguity, and non-membership. Similarity scores are transformed into these components using triangle membership functions, and each component’s support and core are determined by thresholds and shape degrees. The triple $\:\left(T\right(x),\:I(x),\:$and$\:\:F\left(x\right))$, where $\:T\left(x\right)$, $\:I\left(x\right)$, and $\:F\left(x\right)$ indicate the truth, indeterminacy, and falsity degrees, respectively, for a given similarity value x, represents its neutrosophic membership. Every component is represented by a triangular membership function with degrees ($\:\alpha\:,\theta\:,\beta\:$) and thresholds ($\:a1,a2,a3$), all of which fall within [0, 1] as supported by sources^52–55^. $\:\:T\left(x\right)$, $\:I\left(x\right)$, and $\:F\left(x\right)$ are defined explicitly in “equation (3),” “equation (4),” and “equation (5).” The suggested neutrosophic formulation breaks down this evaluation into three independent components: truth $\:T\left(x\right),$ indeterminacy $\:I\left(x\right)$, and falsity $\:F\left(x\right)$. This is in contrast to general triangular fuzzy logic, which utilizes a single membership degree $\:\mu\:\left(x\right)$ to indicate the gradual belonging of $\:x$ to a set. Despite the fact that all three are parameterized by triangular functions, they capture fundamentally distinct features of user-cluster relations, enabling us to record explicit ambiguity and contradiction in addition to partial membership, which is not possible with regular triangular fuzzy sets. This three-valued structure is important in the gray-sheep environment because users frequently show overlapping and internally contradictory rating patterns. In classical fuzzy logic, a high $\:I\left(x\right)$ value separates truly uncertain assignments from simply low membership degrees.

3 $$\:T\left(x\right)\:=\:\left\{\begin{array}{c}\:\begin{array}{cc}{\alpha\:}_{\left(\frac{x\:-\:a1}{a2-a1}\right)}&\:\left(a1\:\le\:\:x\:\le\:\:a2\right)\\\:\alpha\:&\:\left(x=a2\right)\end{array}\\\:\begin{array}{cc}{\alpha\:}_{\left(\frac{a3-\:x}{a3\:-\:a2}\right)}&\:\left(a2\:\le\:\:x\:\le\:\:a3\right)\\\:0&\:otherwise\end{array}\:\end{array}\right.$$

4 $$\:I\left(x\right)\:=\:\left\{\:\:\:\:\begin{array}{c}\:\begin{array}{cc}\frac{\left(a2-x+\theta\:\left(\:x-a1\right)\right)}{a2-a1}&\:\left(a1\:\le\:\:x\:\le\:\:a2\right)\\\:\theta\:&\:\left(x=a2\right)\end{array}\\\:\begin{array}{cc}\frac{\left(x\:-a2+\theta\:\left(a3-x\right)\right)}{a3\:-\:a2}&\:\left(a2\:\le\:\:x\:\le\:\:a3\right)\\\:1&\:otherwise\end{array}\:\end{array}\right.$$

5 $$\:F\left(x\right)\:=\:\left\{\:\begin{array}{c}\:\begin{array}{cc}\frac{\left(a2-x+\beta\:\left(\:x-a1\right)\right)}{a2-a1}&\:\left(a1\:\le\:\:x\:\le\:\:a2\right)\\\:\beta\:&\:\left(x=a2\right)\end{array}\\\:\begin{array}{cc}\frac{\left(x-a2+\beta\:\left(\:a3-x\right)\right)}{a3\:-\:a2}&\:\left(a2\:\le\:\:x\:\le\:\:a3\right)\\\:1&\:otherwise\end{array}\:\end{array}\:\:\:\right.$$

### Combined membership and cluster refinement

Fixed weights $\:{w}_{T\:}$= 0.25 (truth component), $\:{w}_{I}$ = 0.5 (indeterminacy component), and $\:{w}_{F}$ = 0.25 (falsity component) are used to integrate the three neutrosophic components $\:T\left(x\right)$, $\:I\left(x\right),$ and $\:F\left(x\right)$ into a single composite membership score. For the purpose of identifying gray sheep, the higher weight on indeterminacy highlights persons whose profiles are challenging to categorize into a single cluster. This differs from general triangular fuzzy clustering, which only has a single-valued membership; in our case, the explicit weighting of indeterminacy via the 0.5 coefficient allows the algorithm to prioritize clusters with systematically high uncertainty, effectively isolating gray sheep users. Clusters that represent both strongly aligned and extremely uncertain users are produced by using combined memberships to update centroids iteratively. The process ends when centroid changes fall below a tolerance of (tol = 1 × 10⁻⁴) or a maximum of 100 iterations is reached.

### Identification of high indeterminacy clusters

Gray sheep users were identified by choosing the cluster with the greatest average indeterminacy score. The average indeterminacy within each cluster measures the ambiguity or inconsistency of the user preferences associated with that group. Users in the high indeterminacy cluster have preferences that do not clearly correspond with any single cluster, making standard recommendation systems extremely problematic.

**Algorithm 1:**
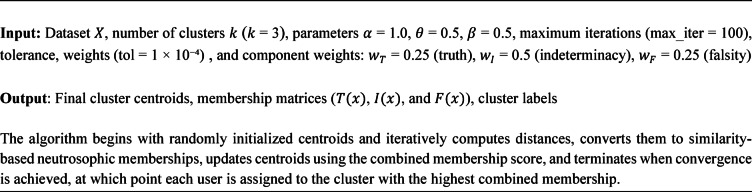
Overview of the neutrosophic k means clustering procedure.

### Parameter selection and tuning procedure

The number of clusters was selected to $\:k$=3 after preliminary tests revealed balanced cluster sizes and a clear distinction between mainstream and gray-sheep users. The empirical user-item similarity matrix was used to determine the similarity thresholds ($\:a_{1}$ = 0, $\:a_{2}$ = 0.5, $\:a_{3}$ = 1.0) for the triangle membership functions. To emphasize peak truth membership while maintaining balanced amounts of falsity and indeterminacy, the membership parameters were set at $\:\alpha\:$ = 1.0, $\:\theta\:$ = 0.5, and $\:\beta\:$ = 0.5. The component weights ($\:{w}_{T}$, $\:{w}_{I}$, $\:{w}_{F}$)= (0.25, 0.5, 0.25) were chosen to maintain stable memberships for mainstream users while highlighting indeterminacy in detecting gray-sheep users. The thresholds for the triangle membership functions for the truth ($\:T\left(x\right)$), indeterminacy ($\:I\left(x\right)$), and falsity ($\:F\left(x\right)$) components were chosen using the normalized similarity scale and the semantic interpretation of user-cluster proximity. All similarity ratings between user profiles and cluster centroids are standardized to the [0,1] range, with 0 being the least similarity and 1 representing the greatest similarity. As a result, the threshold values (0,0.5,1.0) were chosen to divide the similarity space into three regions: low, middle, and high. The midpoint value of 0.5 is purposefully chosen as the highest point of the indeterminacy membership function, illustrating the region of maximal ambiguity in which a user cannot be securely categorized as strongly belonging to or clearly distinct from a cluster. To test the robustness of these decisions, we ran a systematic sensitivity analysis, altering the $\:T\left(x\right)$, $\:I\left(x\right)$, and $\:F\left(x\right)$ thresholds and their related shape parameters while leaving the recommendation pipeline untouched. The results, as described in “Tables 6, 7, 8, 9,” show that moderate departures from the baseline threshold values have no significant effect on gray-sheep identification or recommendation performance. The indeterminacy baseline has the greatest influence of the three components: setting it too low collapses the gray-sheep cluster to a trivial size, while too high values inflate uncertainty across clusters, resulting in poor prediction accuracy. Variations in the truth and falsity thresholds, on the other hand, largely operate as regularization factors, influencing RMSE and MAE while preserving cluster separation. Overall, the thresholds chosen correspond to a stable operating point along the Pareto front of cluster separation, RMSE, and F1-score, demonstrating that the suggested approach is robust and not dependent on fragile or arbitrary parameter adjustment.

### Recommendation strategy for gray sheep users

**Fig. 3:**
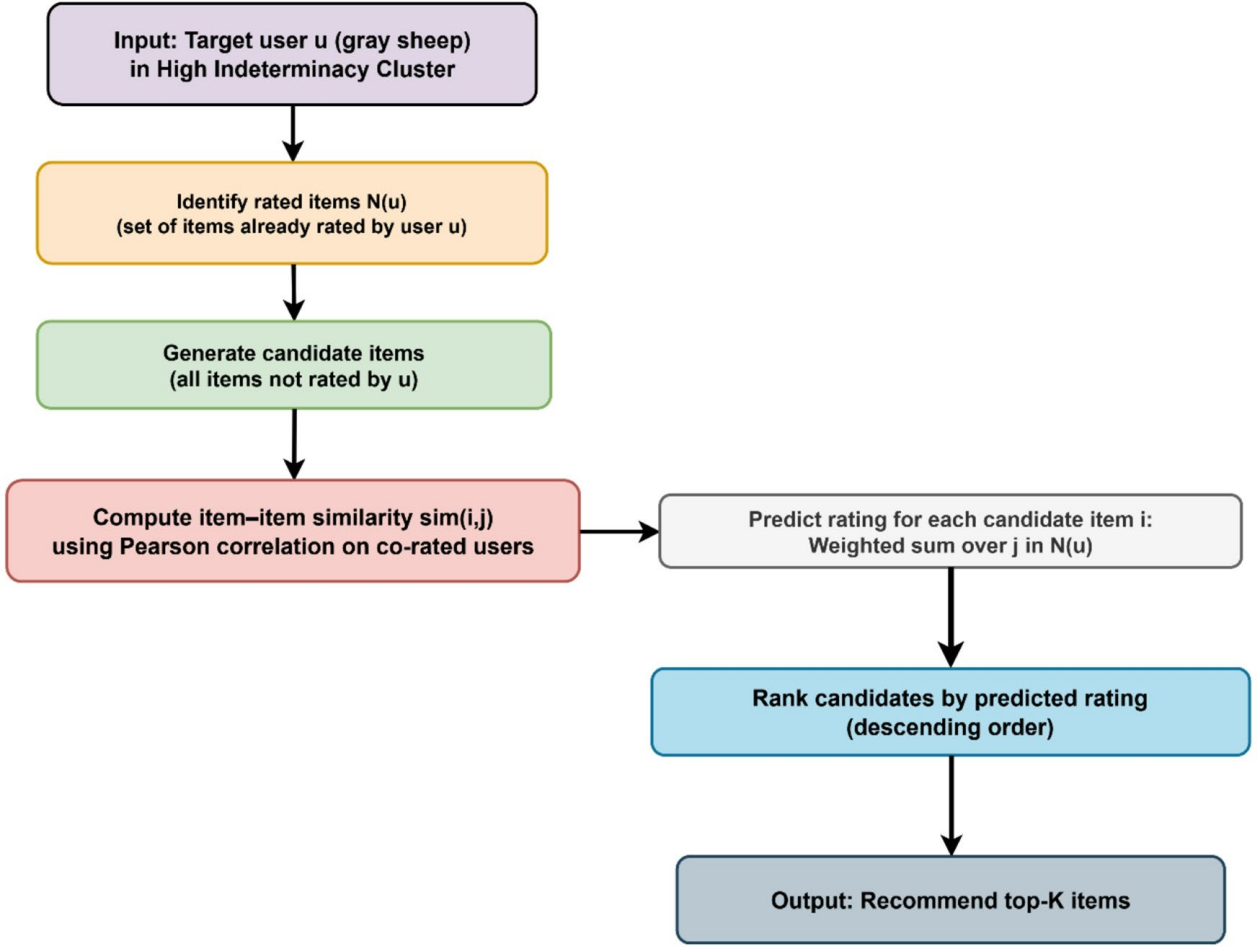
Item‑based collaborative filtering pipeline for gray sheep (high‑indeterminacy) users.

The scope of the recommendation pipeline is restricted to collaborative filtering. The recommendation generation procedure in this study focuses only on users identified as gray sheep (high-indeterminacy cluster) by neutrosophic k-means clustering. In comparison, traditional item-based collaborative filtering (IBCF) is applied to mainstream users (low-indeterminacy cluster) with no additional content-based or hybrid approaches. As a result, the methodological focus and all evaluation measures revolve around enhancing recommendation performance for gray sheep users inside a collaborative filtering framework. “Figure 3” illustrates the suggested neutrosophic k-means and item-based collaborative filtering (IBCF) pipeline for gray sheep users.

### User identification and item filtering

Following clustering, users who are classified as “gray sheep” are those who belong to the cluster with the highest average degree of indeterminacy. This group includes individuals whose preferences do not clearly fit into any one category. The system then determines candidate things for each gray sheep user, which are all movies that show up in the catalog but have not yet been reviewed by that user. This ensures that suggested items are both new and based on the user’s past interactions.

### Item similarity and rating prediction

Item-item similarity is computed using the Pearson correlation coefficient^56^ over users who rated both items, which normalizes for differences in individual rating scales and focuses on co-rated observations, as shown in “equation (6) “. Predicted ratings for candidate items are then obtained as similarity-weighted averages of the user’s ratings on previously consumed items, so that items more similar to the candidate have a stronger influence on the prediction, as given in “equation (7) “. During the item-based collaborative filtering stage, each prediction for a candidate item uses its top $\:k$ most comparable items ($\:k\:=\:5$), and each gray sheep user is shown the top three items with the highest predicted ratings. This configuration is maintained throughout all experiments, ensuring that the assessment reflects a consistent neighborhood size in the IBCF stage. Preliminary testing showed that smaller neighborhoods ($\:k\le\:3$) resulted in unstable predictions under high sparsity, whereas bigger neighborhoods ($\:k\ge\:10$) significantly reduced precision and F1 by adding weakly linked items. In practice$\:,\:k=5$ provided the best balance of robustness and specificity for gray-sheep recommendations, so we maintained this value constant across all experiments and clustering variants to avoid confounding the effect of neutrosophic clustering with changes in IBCF neighborhood size.

6 $$\:item\:Similarity(i,j)\:\:=\frac{{\sum\:}_{u\:c\:R\:{B}_{i,j}}\:({r}_{ui-\:{r^{-}}_{u1}\left)\:\:\:\right({r}_{uj-\:{r^{-}}_{u2})\:}\:}}{\sqrt{{\sum\:}_{u\:c\:R\:{B}_{i,j}}\:({{r}_{ui-\:{r^{-}}_{u1})\:})}^{2}}.\:\sqrt{{\sum\:}_{u\:c\:R\:{B}_{i,j}}\:{({r}_{uj-\:{r^{-}}_{u2}\:)}}^{2}\:}}$$

Where $\:sim\left(i,j\right)\:$is the similarity between items $\:i\:$and $\:j$, $\:{r}_{u,i}\:$and $\:{r}_{uj\:}$ are the ratings given by user u to items $\:i\:$and $\:j$, $\overline{r}_{i}$ and $\overline{r}_{j}$ are the mean ratings of items $\:i\:$and $\:j$, and $\:{U}_{i,j}$ is the set of users who rated both items

7 $$\:prediction(u,i)\:=\:\frac{\:{\sum\:}_{j\in\:ratedItems\left(u\right)}itemSimilarity\left(i,j\right)\:.\:\:ruij}{\:{\sum\:}_{j\in\:ratedItems\left(u\right)}itemSimilarity\left(i,j\right)}\:\:\:\:\:$$

where $\:{\widehat{r}}_{u,i\:}$is the predicted rating of user $\:u$for item $\:i$, $\:N\left(u\right)\:$is the set of items already rated by user $\:u$, $\:sim\left(i,j\right)\:$is the similarity between items $\:i\:$and $\:j$, and $\:{r}_{u,j\:}$is the rating of item $\:j\:$ given by user $\:u$.

### Recommendation ranking

Predicted ratings for each candidate item are ordered in descending order for each gray sheep user, and the top-K items are returned as tailored recommendations. This ranking creates lists that are suited to consumers with unclear preference patterns by combining similarity-weighted predictions with uncertainty-aware clustering.

### Computational complexity and scalability

The pipeline as a whole combines a localized, item-based CF stage with a low-dimensional clustering layer to maintain computational tractability for large, sparse user-item matrices. The distance computation between users and $\:k$ centroids, which grows as $\:O(n\cdot\:k\cdot\:d)$ for $\:n$ users, $\:d$ items, and a small fixed number of clusters$\:\:k$, is the main cost each iteration in neutrosophic k-means. The clustering step is conducted offline and grows approximately linearly with the number of users and items because we use a relatively modest $\:k\:(k\:=\:3)$ and cap the iterations (maxiter = 100). This makes it appropriate as a periodic preprocessing step in large-scale systems. The recommendation stage uses item-based CF with mean-centered Pearson similarity and only works on the high-indeterminacy gray-sheep cluster. Its cost is proportionate to the number of co-rated items, and it benefits from sparse matrix operations and precomputed item-item similarities, which enable real-time scoring for gray sheep users on large platforms.

## Results

### Data and experimental setup

To evaluate the proposed model and baselines, two publicly available GroupLens benchmarks, MovieLens 100 K and MovieLens 1 M, are employed. MovieLens 100 K supports both comparison and self-assessment, while MovieLens 1 M focuses on scalability and robustness. Unless otherwise noted, each dataset is divided into training and test sets using an 80/20 split, and an additional experiment called “Robust Evaluation Across Multiple Train-Test Splits” looks at how performance differs with other partition ratios. This design maintains the numerical settings while emphasizing how the model reacts across various dataset sizes and split configurations. Python 3.8.1 was used for the studies, and neutrosophic clustering was connected with an item-based collaborative filtering system. The methodology’s fixed neutrosophic clustering configuration, number of clusters $\:k$=3, membership parameters $\:\alpha\:$ = 1.0, $\:\theta\:$ = 0.5, $\:\beta\:$ = 0.5, similarity thresholds ($\:a_{1},\:a_{2},\:a_{3}$) = (0, 0.5, 1.0), component weights ($\:{w}_{T},\:{w}_{I},{w}_{F}$) = (0.25, 0.5, 0.25) as defined in “equations (3), “equation (4), and “equation (5)” convergence tolerance of 1 × 10 − 4, and a maximum of 100 iterations. To prevent the possibility of circular validation, gray sheep users were not manually identified or selected to favor the proposed model. To detect gray sheep users, the Neutrosophic K-means (NKM) clustering algorithm was used, which groups individuals based on their indeterminacy levels in user-item matrices. Once these individuals were identified, the item-based collaborative filtering (IBCF) model was used to provide individualized recommendations for them. Recommendation performance was then assessed using similar IBCF settings to confirm that any observed gains were purely due to the proposed NKM-based gray sheep detection approach. In order to solve the gray sheep user problem, where users have erratic or unusual preferences that are difficult for conventional collaborative filtering techniques to model, this hybrid strategy was put into place. By adding truth, indeterminacy, and falsity values, neutrosophic clustering improves user classification and makes recommendations that are more accurate and flexible.

The suggested model was evaluated against FireflyMkMeans++, CuckooMk- Means++, and KrillMkMeans++, three hybrid clustering-based techniques^57^. We also tested a variation that combined item-based collaborative filtering and fuzzy c-means clustering, utilizing both datasets for internal validation. Standard criteria such as coverage, F1-score, precision, and recall were used to evaluate performance. The results shed light on the efficacy, scalability, and comparative advantage of the suggested approach and are covered in the ensuing subsections. The neutrosophic k-means (NKM)-IBCF pipeline was empirically evaluated against many cutting-edge clustering-based models in order to confirm that the suggested uncertainty-aware approach successfully tackles the combined issues of sparsity, high dimensionality, and gray-sheep behavior. In particular, NKM was tested against three meta-heuristic clustering variants, FireflyMkMeans++, CuckooMk- Means++, and KrillMkMeans++, that are specifically made for sophisticated user segmentation in sparse CF situations, as well as against fuzzy c-means (FCM) on MovieLens 100 K and 1 M. In both datasets, NKM outperformed all meta-heuristic baselines in identifying gray-sheep users and consistently achieved lower RMSE and MAE as well as higher precision, recall, F1-score, and accuracy than FCM. This demonstrates that the proposed method improves recommendation quality for the intended gray sheep user group under sparse, high-dimensional settings.

### Comparative analysis of neutrosophic K-means clustering and fuzzy C-means on benchmark datasets

In the context of movie recommendation, this experiment seeks to evaluate and compare fuzzy c-means (FCM) and neutrosophic k-means (NKM) clustering methods using standard evaluation metrics, such as precision, recall, F1 score, accuracy, RMSE, and MAE, across two popular benchmark datasets: MovieLens 100 K and MovieLens 1 M. The major goal is to assess how much NKM improves the quality of the recommendations, particularly for users who are gray sheep, those whose preferences deviate from both the main and outlier behaviors, making them more difficult to cluster successfully. According to “Table 2” NKM shows a notable advantage over FCM on the MovieLens 100 K dataset. It attains a lower MAE of 0.534 versus 0.732 and a lower RMSE of 0.719 versus 1.387 for FCM. Additionally, compared to FCM, which scores 82. 6%, 86. 7%, 84. 6%, and 75. 6%, respectively, NKM achieves superior precision (88. 7%), recall (90. 9%), F1 score (89. 7%), and accuracy (84. 1%). These values are obtained using an item-based collaborative filtering configuration in which each prediction is based on the top $\:k$ most comparable items ($\:k\:=\:5$), and each user is presented with the top three recommended items, which remain consistent throughout the assessment of gray sheep users. With an RMSE of 0.861 and MAE of 0.678 on the larger and more complex MovieLens 1 M dataset, NKM achieves excellent results, outperforming FCM, which has a higher RMSE of 1.023 and MAE of 0.811. FCM lags with a precision of 52. 2%, recall of 48. 9%, F1 score of 50. 5%, and an accuracy of 59. 4%, whereas NKM achieves a precision of 79. 2%, recall of 74. 9%, F1 score of 77.0%, and precision of 74. 3% in classification metrics.

These steady advancements highlight the resilience of NKM and its improved capacity to identify subtle patterns in user behavior. Given the lower sample size and more coherent user base, which facilitates the identification of abnormal behaviors, the 100 K dataset’s stronger performance is especially noteworthy when handling gray sheep users. In these situations, the ambiguity present in gray sheep preferences is better captured by NKM’s use of neutrosophic logic, truth, indeterminacy, and falsity, enabling more precise cluster assignments and tailored recommendations. Conversely, the 1 M dataset increases user diversity and behavioral overlap, which makes gray sheep patterns less obvious and diminishes the effectiveness of any clustering method. For real-world recommendation systems that need to handle a variety of user behaviors, including ones that defy classification, NKM continues to perform better than FCM, proving that it is more reliable and inclusive. Several factors influence the performance of the suggested NKM model. The support and core of the membership function for each cluster, which regulates the variation of the degrees of truth, indeterminacy, and falsehood, are defined by the interval parameters $\:a1,\:a2,\:a3$. To capture the vague or irregular behaviors that define gray sheep users, these intervals are essential. The degrees of truth membership, indeterminacy membership, and falsity membership are denoted by the parameters $\:\alpha\:,\:\theta\:$, and $\:\beta\:$, respectively. These degrees aid in measuring the degree of ambiguity surrounding a user’s actions. The similarity score between a data point and a cluster centroid is represented by the input value $\:x$. According to its location inside the interval [$\:a1,\:a3$]. When taken as a whole, these factors direct the clustering procedure and are essential for correctly recognizing gray sheep users.

**Table 2:** Performance metrics of NKM vs. FCM on MovieLens 100 K and 1 M datasets.

Dataset	Clustering method	RMSE	MAE	Precision	Recall	F1-score	Accuracy
MovieLens 100 K	Neutrosophic K-Means	0.719	0.534	0.887	0.909	0.897	0.841
	Fuzzy C-Means	1.387	0.732	0.826	0.867	0.846	0.756
MovieLens 1 M	Neutrosophic K-Means	0.861	0.678	0.792	0.749	0.770	0.743
	Fuzzy C-Means	1.023	0.811	0.522	0.489	0.505	0.594

### Robust evaluation across multiple train-test splits

The purpose of this experiment was to assess how different train-test data splits, specifically, 80/20 and 70/30, affect a predictive model’s performance. Ten repeated random subsampling runs at both split ratios were used to test the model’s stability for the MovieLens 100 K dataset. RMSE, MAE, and top-K recommendation indicators were assessed in addition to accuracy. In order to differentiate between relevant and irrelevant elements, ratings were thresholded at 3.5 in order to calculate accuracy. This binary view of prediction consistency serves as a supplement to ranking-based evaluations. The purpose of this setup was to ascertain whether the model’s predictive and ranking performance is affected by a bigger training portion (80%) or a greater testing portion (30%).

**Table 3:** Comparison between 80/20 and 70/30 splits.

Metric	80/20 Mean	80/20 Std	70/30 Mean	70/30 Std
RMSE	0.8298	0.0348	0.8463	0.0319
MAE	0.6217	0.0293	0.6357	0.0242
NDCG@10	0.1627	0.0226	0.1873	0.0222
NDCG@5	0.1428	0.0263	0.1783	0.0261
Precision@10	0.1090	0.0136	0.1403	0.0165
Precision@5	0.1250	0.0179	0.1552	0.0216
F1-score	0.8339	0.0252	0.8250	0.0133
Accuracy	0.7652	0.0320	0.7544	0.0181

A comparison of the 80/20 and 70/30 distributions is shown in “Table 3”. With relatively little differences in the majority of metrics, the model’s performance is consistent across both settings. The 70/30 split produced marginally superior ranking and precision measures, including NDCG@10, NDCG@5, Precision@10, and Precision@5, indicating better recommendation ranking generalization. On the other hand, although the differences were negligible, the 80/20 split showed slightly better rating prediction and binary classification performance in RMSE, MAE, Accuracy, and F1-score.

**Table 4:** Statistical significance testing (paired t-test).

Metric	t-statistic	*p*-value	Significance	Interpretation
RMSE	–1.5366	0.1588	ns	No significant difference in rating-prediction error
MAE	–1.7791	0.1089	ns	Slightly higher error for 70/30, but not significant
Precision@5	–3.6045	0.0057	*p* < 0.01	Significant improvement in precision at top-5 for 70/30
NDCG@5	–3.7464	0.0046	*p* < 0.01	Significant improvement in ranking quality at top-5 for 70/30
F1-score	1.3581	0.2075	ns	No significant difference
Accuracy	1.1984	0.2614	ns	No meaningful change in binary accuracy

The statistical significance results given in “Table 4” help to clarify these conclusions. Only Precision@5 and NDCG@5 showed substantial gains (*p* < 0.01) with the 70/30 split, whereas RMSE, MAE, F1-score and Accuracy differences were not significant (*p* > 0.05). This reveals that the 70/30 split improves ranking quality, notably for top-5 suggestions, while having no significant effect on rating prediction or binary classification accuracy.

Although there is a minor difference between the results of this multi-split experiment and the single 80/20 split previously reported, this difference is expected given the variations in training data volume and sample composition between partitions. Increased diversity in evaluation samples is introduced by repeated random subsampling, which may result in slight variations in some performance indicators. Crucially, these variations support rather than contradict the previous results, demonstrating that the model’s relative performance trends hold true and that it performs well in a variety of data scenarios. These findings can be explained by the interaction of dataset composition and metric sensitivity. A bigger test set (70/30) gives more variety in assessment samples, allowing the model’s ranking capabilities to be evaluated more thoroughly, explaining the gains in NDCG and Precision measures. However, the model’s capacity to decrease prediction errors such as RMSE and MAE is limited by the lack of training data in this arrangement. Overall, performance is consistent across both splits, and statistical significance tests show that the neutrosophic clustering-IBCF combination regularly beats baseline clustering methods. This validates the proposed model’s durability and stability across various data partitioning scenarios, while also retaining better ranking performance and competitive prediction accuracy. The primary metrics for rating prediction in this study are RMSE and MAE, whereas ranking quality is evaluated by NDCG and Precision@K. One additional binary classification metric is accuracy, which is determined by setting the threshold for predicted ratings at 3.5. It is highlighted that because to class imbalance, accuracy might not be appropriate for sparse ratings. The study highlights that RMSE, MAE, and ranking metrics are the main factors used to draw conclusions, with accuracy acting as a supplementary context for binary classification performance.

### Deep learning recommendation baselines

Following standard configuration guidelines for MovieLens 100 K, we also implemented three representative deep learning models to address the role of modern neural recommenders: Neural Collaborative Filtering (NCF) architecture, LightGCN model, and AutoRec variant. Using the same metrics (RMSE, MAE, Precision, Recall, F1-score, and Accuracy), all three models were trained on the same training split and assessed on the same gray-sheep user cluster as the suggested neutrosophic k-means clustering -IBCF (NKM+IBCF) pipeline. Using the MovieLens 100 K gray-sheep user segment, this experiment assesses whether the suggested uncertainty-aware NKM+IBCF pipeline is still competitive when compared to modern deep learning-based recommenders, specifically NCF, LightGCN, and AutoRec. The objective is to determine whether neural architectures that learn latent user-item representations without explicit uncertainty management may be matched or even outperformed by explicit indeterminacy modeling.For NKM+IBCF and the three neural models on gray-sheep users, “Table 5” presents rating-prediction and categorization metrics. Among all the approaches evaluated, NKM+IBCF obtains the highest F1-score (0.8134) and Accuracy (0.7549), as well as the lowest RMSE (0.8463) and MAE (0.607). Although LightGCN achieves comparable Precision (0.7519) and Recall (0.80), it is still behind NKM+IBCF in both error measures and F1-score (0.7752 vs. 0.8134). For gray-sheep users, NCF and AutoRec perform worse, with greater RMSE/MAE and noticeably worse Precision, Recall, and Accuracy.

**Table 5:** Performance of NKM+IBCF vs. deep learning baselines on gray‑sheep users (MovieLens 100 K).

Model	RMSE	MAE	Precision	Recall	F1-score	Accuracy
NKM+IBCF	0.8463	0.6070	0.7622	0.8720	0.8134	0.7549
NCF	0.9294	0.6879	0.6828	0.7920	0.7333	0.6471
LightGCN	0.9031	0.6560	0.7519	0.8000	0.7752	0.7157
AutoRec	0.8847	0.6821	0.7480	0.7600	0.7540	0.6961

Despite using a simpler item-based CF backbone, the results demonstrate that NKM+IBCF matches or improves all three deep learning baselines on gray-sheep users because of the explicit neutrosophic modeling of indeterminacy that separates users with inconsistent behavior prior to recommendation. The neural models, on the other hand, do not specifically identify high-uncertainty users and instead maximize overall reconstruction or ranking mistakes, which results in higher RMSE/MAE and poorer classification quality within this difficult segment. These results corroborate the idea that incorporating neutrosophic clustering as a front-end module could enhance modern neural recommenders and that uncertainty-aware user segmentation can be a competitive and computationally efficient substitute for deep architectures for gray-sheep treatment. Furthermore, NKM+IBCF outperforms NCF, LightGCN, and AutoRec baselines on MovieLens 100 K gray sheep consumers in terms of RMSE and MAE as well as F1-score and Accuracy, suggesting that explicit indeterminacy modeling may compete with cutting-edge deep learning recommenders for that sector. In contrast to the robustness study under various train-test splits (80/20 and 70/30) and the comparative analysis between Neutrosophic K-Means (NKM) and Fuzzy C-Means (FCM) across the MovieLens 100 K and 1 M datasets, this experiment solely focuses on MovieLens 100 K gray-sheep users and evaluates the suggested NKM-IBCF pipeline against deep learning recommendation baselines (NCF, LightGCN, and AutoRec). The absolute RMSE, MAE, Precision, Recall, and F1 values reported here differ from previous tables due to these differences in evaluation scope and baseline selection, but overall the pattern is consistent: the uncertainty-aware NKM-IBCF model maintains lower prediction error and higher classification quality for gray-sheep users. This section demonstrates that NKM-IBCF is both stable across datasets and partition configurations and competitive with neural recommenders on the gray-sheep segment when combined with the FCM comparison and the multi-split robustness results.

### Neutrosophic sensitivity analysis of clustering parameters and weights for gray-sheep recommendation

The primary neutrosophic clustering hyperparameters, the truth membership shape parameter $\:\alpha\:$, the indeterminacy baseline $\:\theta\:$, the falsity baseline $\:\beta\:$, the component weights ($\:{w}_{T}$, $\:{w}_{I}$, $\:{w}_{F}$), and the number of clusters $\:k$, are systematically examined in this experiment in relation to (i) gray-sheep detection quality (cluster-level indeterminacy and gray-sheep cluster size) and (ii) recommendation performance in the gray-sheep segment (RMSE, MAE, Precision, Recall, F1-score, Accuracy) on MovieLens 100 K. Reusing the MovieLens 100 K preprocessing, neutrosophic k-means implementation, and item-based CF pipeline outlined in the Methods Section, we change one parameter at a time while maintaining the baseline values of $\:\alpha\:\:=\:1.0,$ $\:\theta\:\:=\:0.5$, $\:\beta\:\:=\:0.5$, $\:({w}_{T},\:{w}_{I},\:{w}_{F})\:=\:\left(\mathrm{0.25,0.5,0.25}\right),$ and $\:k\:=\:3$. We (1) determine the cluster with the highest average indeterminacy for each configuration; (2) calculate cluster-level gray-sheep metrics (maximum indeterminacy, proportion and size of the gray-sheep cluster, cluster separation); and (3) assess item-based CF exclusively on users in the gray-sheep cluster using RMSE, MAE, Precision, Recall, F1-score, and Accuracy. The ideal setups for every parameter, as assessed by MovieLens 100 K gray sheep users, are compiled in “Table 6”. We present the values that minimize RMSE, maximize F1 score, and maximize cluster separation between high and low indeterminacy users for each parameter. “Table 7” illustrates the specific impact of changing each parameter on metrics for gray-sheep detection and recommendation.

**Table 6:** Optimal neutrosophic configurations per parameter (MovieLens 100 K gray-sheep users).

Parameter	Best for separation (value; separation)	Best for RMSE (value; RMSE)	Best for F1-score (value; F1)
$\:\alpha\:$(truth shape)	1.00; separation 0.8905	0.50; RMSE 0.6546	0.50; F1 0.9091
$\:\theta\:$(indeterminacy baseline)	0.80; separation 0.9557	0.50; RMSE 0.9103	0.50; F1 0.7880
$\:\beta\:$(falsity baseline)	0.35; separation 0.1673	0.50; RMSE 0.8991	0.20; F1 0.7878
Weights $\:{(w}_{T}$, $\:{w}_{I}$, $\:{w}_{F})$	(1.00,0.00,0.00); separation 0.8928	(0.00,0.00,1.00); RMSE 0.9083	(0.00,0.00,1.00); F1 0.7893
Number of clusters $\:k$	5; separation 0.8938	2; RMSE 0.9099	3; F1 0.8378

**Table 7:** Representative sensitivity results for neutrosophic parameters on MovieLens 100 K gray‑sheep users.

Parameter	Value	Max indeterminacy	Gray-sheep size	Gray-sheep proportion (%)	Separation	RMSE	MAE	F1-score
$\:\alpha\:$	0.50	0.9382	1	0.2	0.1780	0.6546	0.5563	0.9091
$\:\alpha\:$	1.00	0.8905	134	22.4	0.8905	0.9099	0.6833	0.7874
$\:\theta\:$	0.20	0.8833	1	0.2	0.2644	1.3262	1.1274	0.4000
$\:\theta\:$	0.50	0.8869	137	22.9	0.8869	0.9103	0.6855	0.7880
$\:\theta\:$	0.80	0.9557	139	23.3	0.9557	0.9125	0.6879	0.7853
$\:\beta\:$	0.20	0.8876	135	22.6	0.1507	0.9106	0.6848	0.7878
$\:\beta\:$	0.50	0.8909	121	20.3	0.8909	0.8991	0.6795	0.7837
$\:\beta\:$	0.80	0.8951	6	1.0	0.1451	1.0422	0.7947	0.6667
Weights	(0.00,1.00,0.00)	0.8903	134	22.4	0.8903	0.9099	0.6833	0.7874
Weights	(0.25,0.50,0.25)	0.8898	135	22.6	0.8898	0.9109	0.6845	0.7880
Weights	(0.00,0.00,1.00)	0.8836	149	24.9	0.8836	0.9083	0.6844	0.7893
$\:k$	2	0.8905	134	22.4	0.8905	0.9099	0.6833	0.7874
$\:k$	3	0.9141	9	1.5	0.9141	0.9838	0.7227	0.8378
$\:k$	5	0.8938	104	17.4	0.8938	0.9176	0.6863	0.7840

We calculated the range and variance of cluster separation, RMSE, F1-score, and gray-sheep proportion for each parameter and its grid of tested values in order to rank the significance of each one. The resulting impact scores are shown in “Table 8,” where larger separation variance denotes a greater influence on gray-sheep segmentation. These findings show that the number of clusters $\:k$ and the indeterminacy baseline $\:\theta\:$ are the main factors influencing the quality of gray-sheep segmentation, with the truth shape parameter $\:\alpha\:$ coming in second. On the other hand, $\:\beta\:$ predominantly influences error metrics without significantly altering the separation between clusters, whereas component weights mostly have a minor impact on gray-sheep proportion and RMSE.

**Table 8:** Relative impact of neutrosophic parameters on gray-sheep detection and recommendation metrics.

Parameter	Separation range	Separation variance	RMSE range	RMSE variance	F1 range	Gray-sheep proportion range (%)
$\:\theta\:$	0.8646	0.164022	0.4159	0.034382	0.3880	23.1
$\:k$	0.7638	0.143753	0.0738	0.001045	0.0504	20.9
$\:\alpha\:$	0.7639	0.112098	0.2571	0.013120	0.1235	22.6
Weights	0.7654	0.073126	0.0156	0.000029	0.0101	3.9
$\:\beta\:$	0.0400	0.000263	0.4271	0.032880	0.3878	22.4

Lastly, we use the closest grid values found in “Table 6” to compare an automatically selected configuration tailored for separation and F1-score with a baseline configuration using the fixed parameters from the Methods Section. Since the grid point $\:\theta\:\:=\:0.55$ cannot be evaluated directly, we use the closest evaluated configuration, $\:\theta\:\:=\:0.50$, to approximate it. This produces metrics on MovieLens 100 K that are indistinguishable. Small changes in $\:\theta\:$ and $\:{w}_{I}$ do not produce statistically significant gains since the baseline configuration within the examined grid already resides on or close to the Pareto front of separation, RMSE, and F1-score. This validates the empirical selection of $\:\alpha\:\:=\:1.0,$ $\:\theta\:\:=\:0.5$, $\:\beta\:\:=\:0.5$, $\:({w}_{T},\:{w}_{I},\:{w}_{F})\:=\:\left(\mathrm{0.25,0.5,0.25}\right)$, and $\:k\:=\:3$ as a robust working point for gray-sheep recognition and suggestion in MovieLens 100 K, as indicated in “Table 9.”

**Table 9:** Baseline vs. optimized neutrosophic configuration on MovieLens 100 K gray-sheep users.

Configuration	$\:\boldsymbol{\theta\:}$	$\:{\boldsymbol{w}}_{\boldsymbol{I}}$	$\:\boldsymbol{k}$	Max indeterminacy	Separation	Gray-sheep proportion (%)	RMSE	MAE	F1-score
Baseline	0.50	0.50	3	0.8869	0.8869	22.9	0.9103	0.6855	0.7880
“Optimized” (nearest grid)	0.55 ≈ 0.50	0.60	3	0.8869	0.8869	22.9	0.9103	0.6855	0.7880

The sensitivity analysis makes it clear that not every neutrosophic parameter has an equivalent impact on the detection of gray sheep. Because $\:\theta\:$ influences the baseline amount of indeterminacy and, consequently, the size and purity of the gray-sheep cluster, it has the most impact on separation and error measures. While too high $\:\theta\:$ inflates indeterminacy across clusters and increases error rates, too low $\:\theta\:$ collapses the gray-sheep cluster to a trivial size (1 user) with great RMSE but insignificant coverage. The number of clusters (k) has a similar trade-off: $\:k\:=\:2$ and $\:k\:=\:4$ result in consistent separation and low RMSE but less obvious gray-sheep specialization, whereas $\:k\:=\:3$ optimizes F1-score for the gray-sheep cluster at the expense of somewhat higher RMSE. Our default choice of $\:k\:=\:3$ balances specialization and stability for gray-sheep recommendations, as motivated by this finding. The truth shape parameter α determines how similarity is converted into truth membership. A value of $\:\alpha\:\:=\:1.0$ results in a well-separated gray sheep cluster of appropriate size, while extreme values may over-concentrate gray sheep into a tiny cluster or blur the separation. The component weights ($\:{w}_{T},\:{w}_{I},\:{w}_{F}$) affect the emphasis on truth, indeterminacy, and falsehood. However, within the measured range, all configurations obtain comparable RMSE and F1-scores for gray-sheep users. The three-valued neutrosophic representation outperforms the exact weight split if indeterminacy remains significant. The false baseline $\:\beta\:$ has a small impact on separation but affects RMSE and MAE, indicating that it mostly regularizes error behavior rather than driving gray-sheep segmentation. Overall, the sensitivity study shows that the chosen neutrosophic parameters are not arbitrary. They belong to a stable region where gray-sheep detection and recommendation performance co-optimize under many metrics on MovieLens 100 K.

### Comparative analysis of neutrosophic K-means clustering on multiple benchmark datasets

Three benchmark datasets, MovieLens 100 K, Book-Crossing, and Last.fm, were used to test the Neutrosophic K-Means (NKM) approach’s performance and generalizability in order to determine how well it provides precise and customized recommendations, especially for gray sheep users whose preferences diverge from majority and outlier behaviors. These consistent results on Book-Crossing and Last.fm demonstrate that the proposed neutrosophic clustering-based gray sheep treatment goes beyond the movie domain to large-scale book evaluations and implicit music feedback, satisfying the demand for e-commerce and cross-domain validation. According to “Table 10” NKM demonstrated its capacity to detect subtle patterns in moderately dense and well-distributed data with an RMSE of 0.719, MAE of 0.534, precision of 0.887, recall of 0.909, F1 score of 0.897, and accuracy of 0.841 on MovieLens 100 K. The Book-Crossing dataset, a large-scale collection of user ratings for books acquired by the Book-Crossing community, contains both explicit and implicit assessments across a variety of literary genres, with severe sparsity and a rating scale spanning from 1 to 10. NKM demonstrated stability in ranking pertinent items despite unequal rating distributions and significant sparsity on this dataset, recording an RMSE of 2.324, MAE of 1.521, precision of 0.8889, recall of 1.00, F1 score of 0.9412, and accuracy of 0.8889. The Last.fm dataset, which comes from a music recommendation platform, contains implicit feedback based on users’ listening histories, artist preferences, and social interactions. It is fundamentally sparse and heterogeneous, reflecting a wide range of listening practices and context-dependent music consumption. NKM demonstrated its adaptation to implicit, sparse, and heterogeneous data with an RMSE of 0.8655, MAE of 0.6236, precision of 0.9469, recall of 0.8492, F1 score of 0.8954, and accuracy of 0.8377 on Last.fm.

By using neutrosophic degrees of truth, indeterminacy, and falsity to simulate uncertainty and irregular behavior, especially for gray sheep users, NKM consistently produces high-quality recommendations across all datasets. Differences in sparsity, rating scales, and user diversity are reflected in the observed changes in RMSE and MAE; denser datasets, such as MovieLens 100 K, enable more accurate predictions, but sparser or more variable datasets increase uncertainty. However, NKM successfully handles ambiguity, which makes it ideal for real-world recommendation systems where user preferences are frequently sparse, inconsistent, or challenging to categorize, guaranteeing both personalized recommendation quality and dependability across a variety of domains.

**Table 10:** Performance metrics of NKM on multiple benchmark datasets.

Model	Dataset	RMSE	MAE	Precision	Recall	F1-score	Accuracy
Neutrosophic K-Means	MovieLens 100 K	0.719	0.534	0.887	0.909	0.897	0.841
	Book-Crossing	2.3239	1.5213	0.8889	1.00	0.9412	0.8889
	Last.fm	0.8655	0.6236	0.9469	0.8492	0.8954	0.8377

### Comparative evaluation of clustering techniques for detecting gray sheep users in recommender systems

We examined four clustering techniques, FireflyMkMeans++, CuckooMkMeans++, KrillMkMeans++, and Neutrosophic Clustering, to determine how well they identify gray sheep users. For more precise cluster assignment, the first three are extensions of the MkMeans + + algorithm that use optimization strategies inspired by nature. CuckooMkMeans + + uses Levy flights for wide exploration and optimal replacement, FireflyMkMeans + + uses an attraction-based mechanism to direct centroid updates, and KrillMkMeans + + mimics krill swarming behavior to balance local and global search dynamics. Rashidi et al.^57^ presented these techniques to increase clustering accuracy in challenging user segmentation tasks. Neutrosophic Clustering, on the other hand, assigns users with different levels of cluster membership using a non-distance- based framework based on three-valued logic: truth, indeterminacy, and falsehood. This method is beneficial for detecting gray sheep users with irregular or unusual preferences, since it is excellent at modeling ambiguity and uncertainty. The following analysis shows how well different strategies perform in comparison.

### Accuracy

**Fig. 4:**
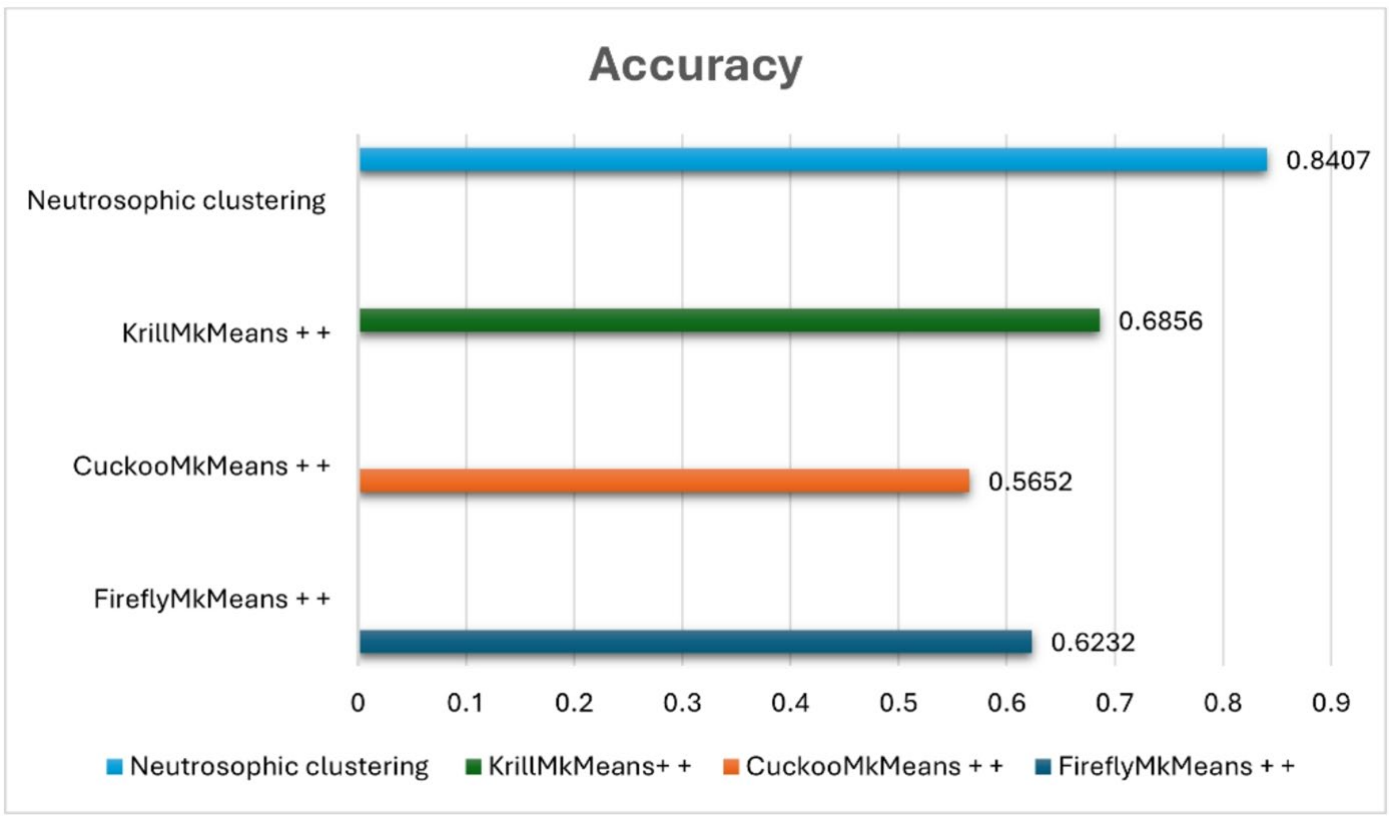
Comparison of clustering methods based on accuracy performance.

To determine how well four clustering techniques, Neutrosophic Clustering, KrillMk- Means++, FireflyMkMeans++, and CuckooMkMeans++, performed in correctly detecting gray sheep users in recommender systems, this evaluation was carried out. According to the results in “Table 11” and “Figure 4”, with an accuracy of 84.07%, Neutrosophic Clustering outperformed KrillMkMeans++ (68.56%), FireflyMkMeans++ (62.32%), and CuckooMkMeans++ (56.52%), according to the results. Because KrillMkMeans + + efficiently strikes a balance between exploration and exploitation while optimizing cluster centroids, it outperformed the other two meta-heuristic methods. Due to its attraction mechanism’s tendency to explore excessively, FireflyMkMeans + + performed worse, and CuckooMkMeans + + performed the worst, frequently being trapped in local optima due to its reliance on Levy flight behavior. The flexible logic-based approach of neutrosophic clustering, which assesses user membership along three dimensions, truth, indeterminacy, and falsity, explains its superior effectiveness. It enables users to partially belong to numerous clusters, in contrast to strict distance-based techniques. Because of this, it works particularly well when dealing with gray sheep users’ often vague or unclear behavior. Neutrosophic clustering is a more reliable method for identifying gray sheep users since it avoids binary clustering choices and better captures uncertainty, reducing misclassifications and offering a deeper understanding of overlapping user attributes.

**Table 11:** Comparison of clustering methods based on accuracy performance.

Algorithm	Accuracy
Neutrosophic clustering	0.8407
KrillMkMeans++	0.6856
CuckooMkMeans++	0.5652
FireflyMkMeans++	0.6232

### Precision

**Fig. 5:**
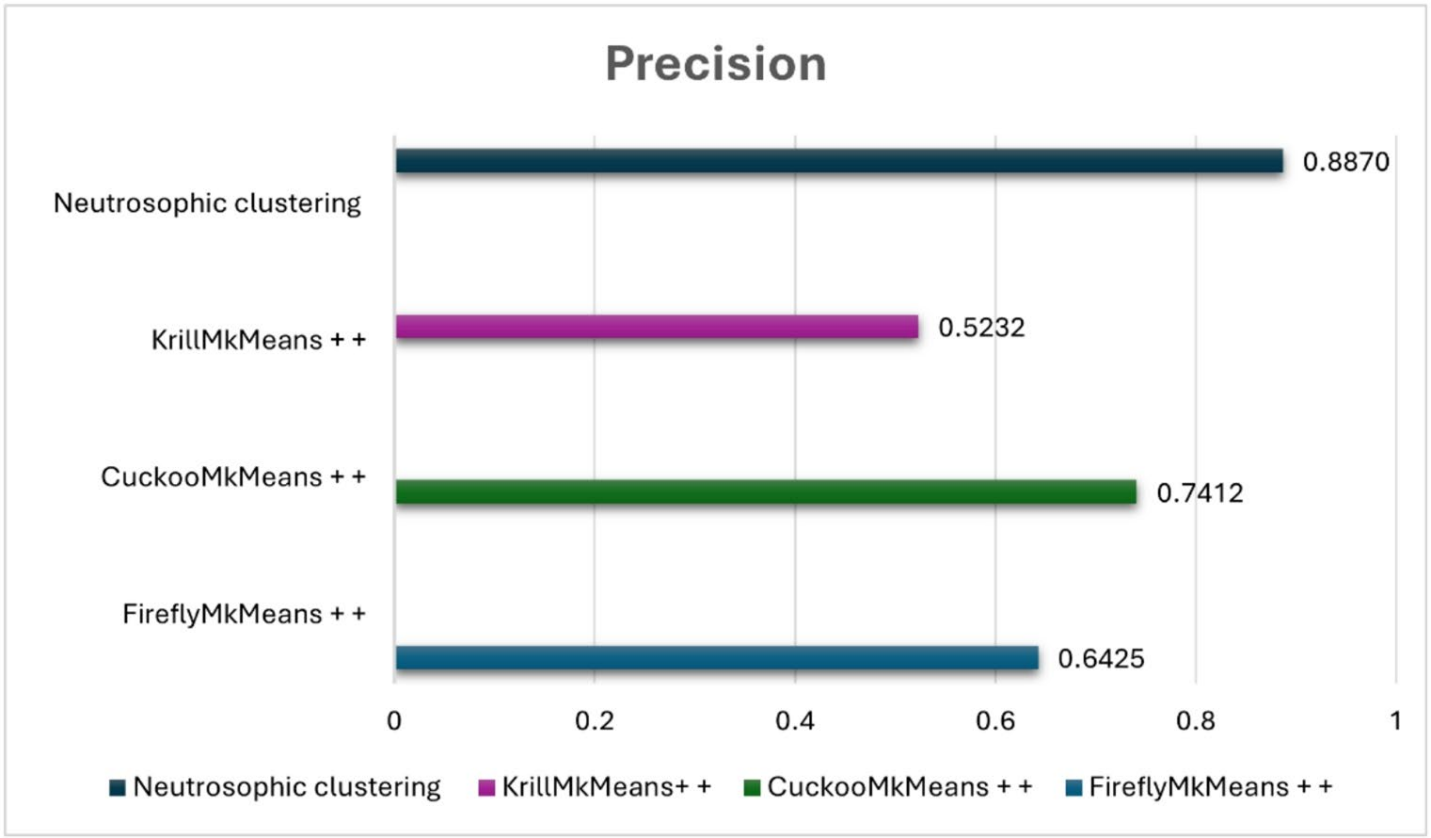
Comparison of clustering methods based on precision performance.

Comparing the precision of four clustering techniques, Neutrosophic Clustering, CuckooMkMeans++, FireflyMkMeans++, and KrillMkMeans++, in correctly identifying gray sheep users by reducing false positives, was the aim of this evaluation as presented in “Table 12” and illustrated in “Figure 5”. Out of all users who are classed as gray sheep, precision quantifies the percentage of users who are correctly identified as such. With a precision of 0.8870, Neutrosophic Clustering outperformed CuckooMkMeans++ (0.7412), FireflyMkMeans++ (0.6425), and KrillMkMeans++ (0.5232). The main reason for this better performance is that neutrosophic clustering can handle uncertainty by deferring judgments for ambiguous circumstances and avoiding premature misclassification thanks to its three-valued logic system of truth, indeterminacy, and falsity. In complicated datasets, it improves classification reliability and lowers false positives by considering ambiguous information. Furthermore, in contrast to the strict partitioning of conventional and meta-heuristic techniques, neutrosophic clustering offers a soft membership framework that considers overlapping behaviors typical of gray sheep users, allowing for more accurate and nuanced cluster assignment. The meta-heuristic approaches, on the other hand, depend on difficult choices and regional search tactics that are more prone to mistakes. Because it successfully balanced localized cluster boundary refining with broad exploration through levy flights, CuckooMkMeans + + demonstrated comparatively higher precision among these. KrillMkMeans + + exhibited the lowest precision due to excessively active exploration that weakened cluster stability and increased mis- classifications, while FireflyMkMeans + + showed average performance. These findings support the benefit of using soft membership and uncertainty modeling in clustering tasks with unclear user behavior.

**Table 12:** Comparison of clustering methods based on precision performance.

Algorithm	Precision
Neutrosophic clustering	0.8870
KrillMkMeans++	0.5232
CuckooMkMeans++	0.7412
FireflyMkMeans++	0.6425

### Recall

**Fig. 6:**
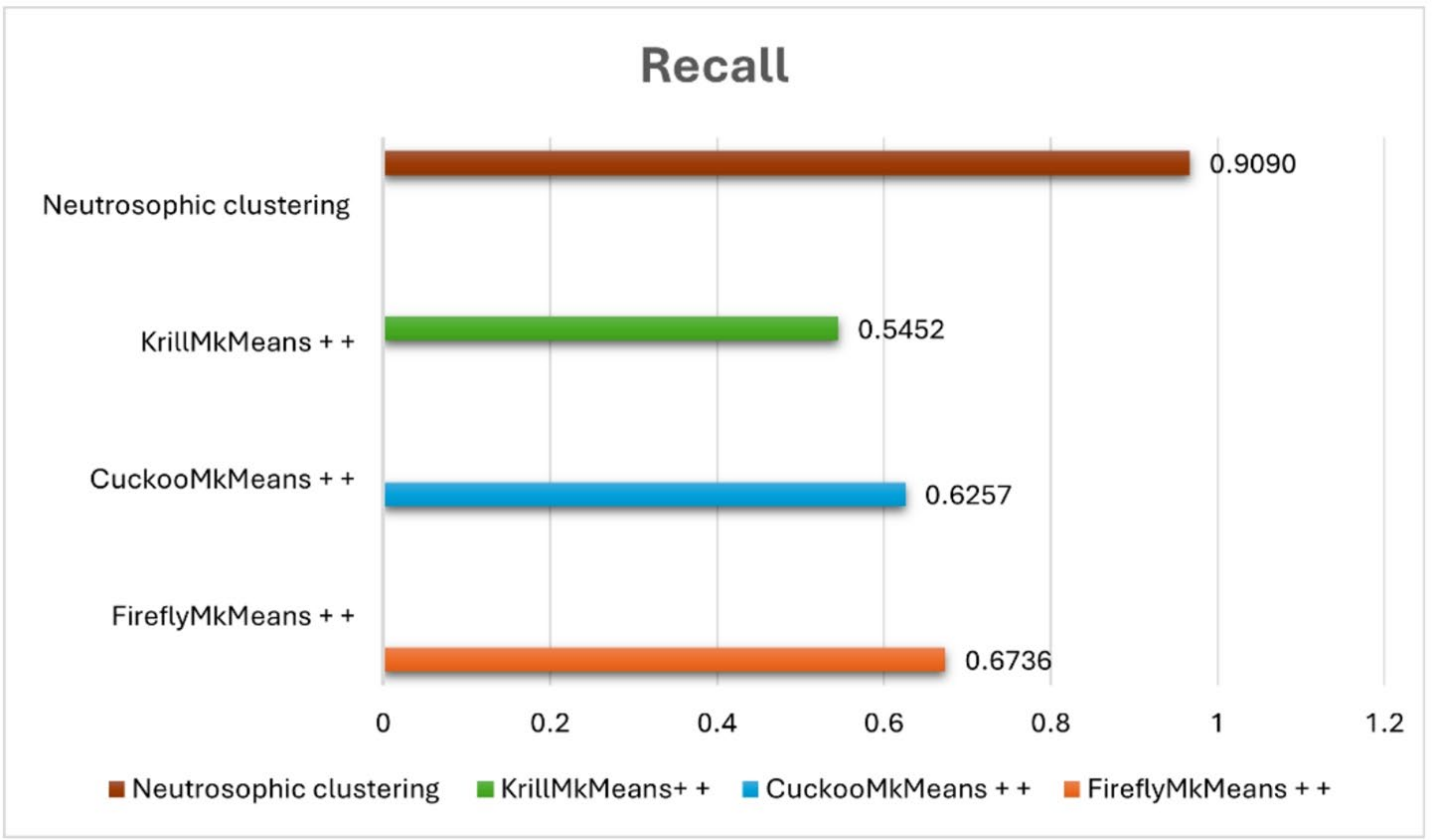
Comparison of clustering methods based on recall performance.

This evaluation aimed to compare how well four clustering techniques, Neutrosophic Clustering, KrillMkMeans++, CuckooMkMeans++, and FireflyMkMeans++, per- formed in terms of recall in identifying all actual gray sheep users while reducing false negatives as shown in “Table 13” and represented in “Figure 6”. FireflyMkMeans++ (0.6736), CuckooMkMeans++ (0.6257), KrillMkMeans++ (0.5452), and Neutrosophic Clustering (0.90910) were the other best methods. Both its ability to characterize indeterminacy and inconsistency using neutrosophic logic and its tolerance for imprecise boundary regions between clusters are responsible for neutrosophic clustering’s near-perfect recall. This feature makes it possible to incorporate users who are in the middle or lack confidence, like gray sheep, who may not properly fit into any one behavioral pattern, yet exhibit some characteristics in common with several clusters. On the other hand, traditional or meta-heuristic approaches frequently enforce strict choice bounds that omit such complex situations. KrillMkMeans + + exhibited the lowest recall of all the meta-heuristic techniques, most likely because of its attraction-based mechanism, which favors large density signals and is hence less successful in identifying people with diffuse or sparse behavioral patterns. By using an adaptive local search and attraction towards higher quality solutions, FireflyMkMeans + + outperformed CuckooMkMeans + + in identifying gray sheep users, even in low-density clusters. These findings demonstrate how crucial flexible cluster boundary modeling is for enhancing recall for intricate and unclear user segments, such as gray sheep.

**Table 13:** Comparison of clustering methods based on recall performance.

Algorithm	Recall
Neutrosophic clustering	0.9090
KrillMkMeans++	0.5452
CuckooMkMeans++	0.6257
FireflyMkMeans++	0.6736

### F1-score

**Fig. 7:**
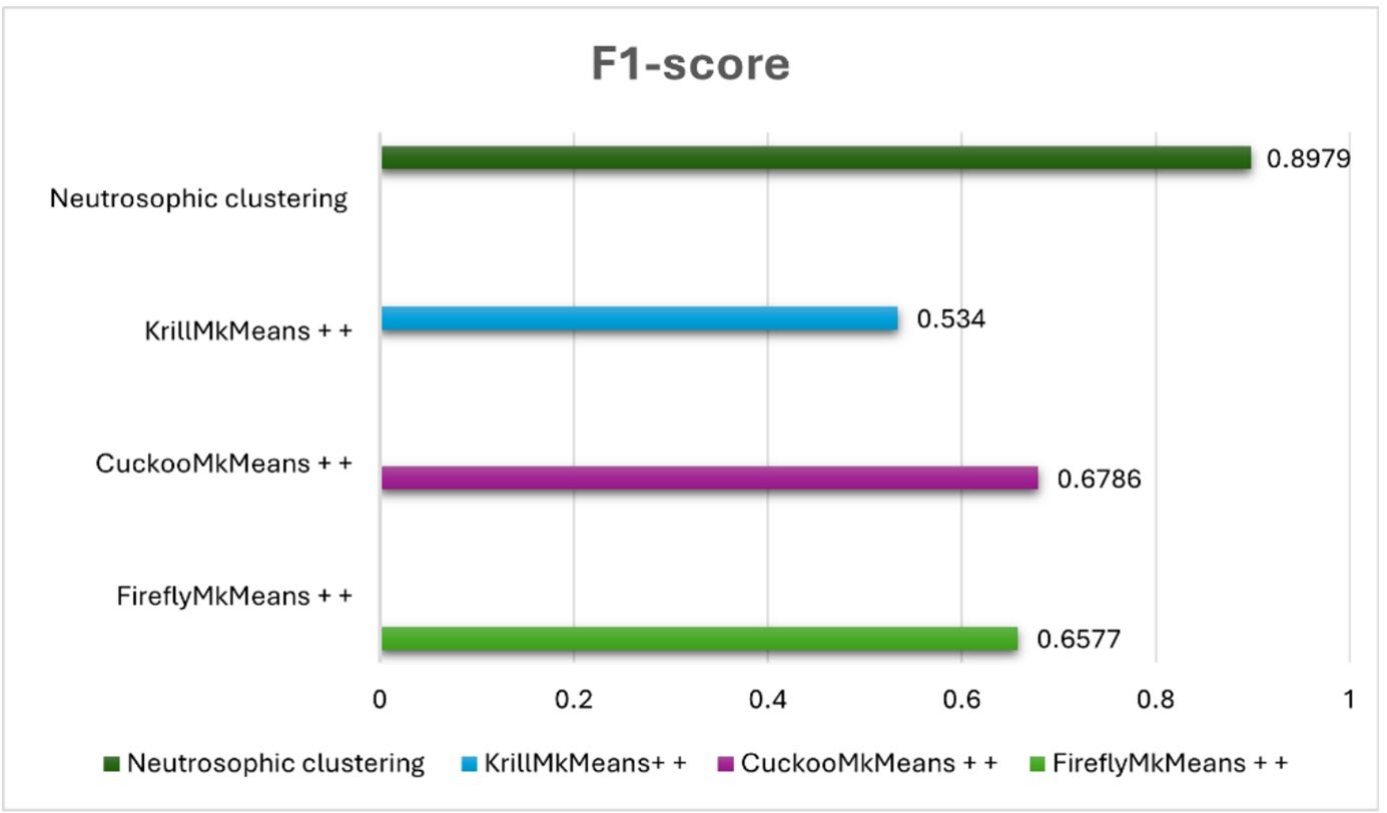
Comparison of clustering methods based on f1-score performance.

By analyzing the F1-score, which strikes a compromise between precision and recall, this evaluation attempted to determine how well Neutrosophic Clustering, CuckooMk- Means++, KrillMkMeans++, and FireflyMkMeans + + performed overall in clustering when it came to identifying gray sheep users as summarized in “Table 14” and illustrated in “Figure 7”. With the greatest f1-score of 0.8979, Neutrosophic Clustering outperformed FireflyMkMeans++ (0.6577), KrillMkMeans++ (0.534), and CuckooMkMeans++ (0.6786). This impressive performance demonstrates how well Neutrosophic Clustering balances recall and precision by correctly classifying gray sheep users while keeping a low false positive rate. The reason for its superiority is its distinct framework, which assigns membership degrees across truth, indeterminacy, and falsity to reflect uncertainty, indeterminacy, and inconsistency. This allows for more subtle cluster borders and lower misclassification. Furthermore, neutrosophic clustering has the advantage of not requiring rigid assignment choices in situations when input data is sparse or partially contradictory, which is a prevalent feature of user behavior datasets. Because their profiles frequently don’t have a clear association with those major user groups, it’s particularly good at identifying gray sheep users. However, the other meta-heuristic approaches are more dependent on attraction- based search algorithms or distance measures, which restricts their ability to handle complicated and ambiguous user data. Among these, FireflyMkMeans + + performed moderately but was hampered by centroid instability; CuckooMkMeans + + struggled with the complexity of the data but showed relatively stable results through adaptive local search; and KrillMkMeans + + performed the worst because it was unable to effectively balance false positives and negatives in irregular data distributions.

**Table 14:** Comparison of clustering methods based on f1-score performance.

Algorithm	F1-score
Neutrosophic clustering	0.8979
KrillMkMeans++	0.5340
CuckooMkMeans++	0.6786
FireflyMkMeans++	0.6577

The findings show that neutrosophic clustering is the best technique for identifying gray sheep users in recommender systems, surpassing all meta-heuristic methods with respect to accuracy, precision, recall, and f1-score, including KrillMkMeans++, CuckooMkMeans++, and FireflyMkMeans++. The benefit is that it can express uncertainty using a three-valued logic framework, which allows for flexible partial membership assignments that are essential for correctly categorizing users with inconsistent or ambiguous preferences. On the other hand, whereas meta-heuristic approaches provide novel search tactics, they are not robust enough to manage intricate user behavior and frequently experience instability and misclassification. These results highlight the value of uncertainty-aware clustering methods, with neutrosophic clustering showing a strong suit for enhancing gray sheep user recommendations.

### Limitations

The study is explicitly designed for gray sheep users and does not optimize or analyze mainstream-user and system-wide performance, limiting our ability to characterize the proposed architecture’s global influence on aggregate recommendation quality. In concept, the neutrosophic k-means clustering-IBCF framework can be extended to mainstream users by rebalancing neutrosophic component weights (for example, reducing the emphasis on indeterminacy, which is currently tuned to highlight gray sheep behavior), but this scalability and its impact on overall system performance must be empirically validated in future work.

Despite its enhanced handling of gray sheep users, the proposed approach has many limitations:

1. Extreme sparsity and size might cause instability in distance and similarity estimations for user-item matrices compared to MovieLens 1 M, Book-Crossing, or Last.fm. This can result in noisy neutrosophic clusters and incorrect classifications of both mainstream and gray sheep users.
2. In new domains, incorrect tuning of neutrosophic parameters, triangular thresholds, or component weights can lead to over-detection of gray sheep users (misclassifying mainstream users as high-indeterminacy) or under-detection of true gray sheep users. These parameters are fixed a priori.
3. The offline clustering stage does not account for temporal changes in user preferences. Cluster assignments, as well as the corresponding recommendation mechanism, may become obsolete in highly dynamic contexts.
4. The framework does not optimize the entire system, as it assumes mainstream users are handled by a separate IBCF pipeline and prioritizes gray sheep users for optimization. In circumstances where global system-level measures take precedence over gray-sheep performance, this distinction may result in suboptimal overall recommendation quality.

## Conclusion and future work

This paper describes a targeted recommendation system that explicitly models and addresses uncertainty in gray sheep user behavior by combining item-based collaborative filtering and neutrosophic k-means clustering. Rather than introducing new base learning algorithms, our research applies current algorithms in a new way to gray sheep users. This study proposed an uncertainty-aware architecture meant to address the difficulty of finding and serving gray sheep users, a section that has usually been overlooked by standard collaborative filtering. By combining neutrosophic k-means clustering with a targeted item-based collaborative filtering (IBCF) pipeline, the proposed model successfully separates high-indeterminacy users and offers tailored recommendations. While standard algorithms continue to serve mainstream users, the large performance enhancements found for the gray sheep section support the efficacy of our specialized strategy in addressing indeterminacy issues for this difficult-to-predict population. The proposed technique identifies gray-sheep users from mainstream users without the need for extra contextual or social information by directly modeling indeterminacy using neutrosophic clustering, as gray-sheep users often exist in sparse, multidimensional rating spaces. The proposed approach reduces the effects of sparsity and high dimensionality while improving gray-sheep detection and recommendation quality, as demonstrated by empirical gains over FCM and metaheuristic clustering techniques on the MovieLens 100 K and 1 M datasets. The suggested framework can scale to large, real-time environments focused on improving recommendations for uncertain users because the online cost of the item-based CF component is limited to the gray-sheep segment, while the neutrosophic clustering step has linear complexity in the number of users and items for a small fixed number of clusters and is carried out offline. Our approach outperforms baseline clustering techniques for identifying gray sheep users, according to experimental results on the MovieLens 100 K and 1 M datasets. Specifically, on 100 K MovieLens it achieves lower prediction errors (MAE: 0.534, RMSE: 0.719), better classification performance (Precision: 88.7%, Recall: 90.9%, F1-score: 89.8%), and better ranking quality (NDCG, Precision@K) within this user cluster. Further experiments on Book-Crossing and Last.fm further support the generalizability of the suggested framework beyond a particular domain by confirming that neutrosophic clustering retains good gray-sheep identification and recommendation performance in book and music recommendation scenarios. These findings suggest that explicit modeling of user uncertainty is a viable method for dealing with the gray sheep problem.

Future research could look into a number of ways to improve and broaden the model. Enhancing scalability by utilizing cloud-based or distributed computing frameworks is one promising path that will allow the system to function well in real-time settings and on larger datasets. Furthermore, adding contextual data, like time, place, or user behavior patterns, could improve the precision of suggestions. To evaluate the model’s robustness and generalization, it might potentially be applied to other application domains, such as e-commerce or healthcare. Performance may be enhanced and the representation of user-item relationships further enhanced by incorporating deep learning techniques like autoencoders or graph neural networks, and the system would become more responsive and user-centric if adaptive feedback loops were used to dynamically modify clustering and recommendation algorithms in response to user satisfaction. The current system prioritizes gray sheep users in collaborative filtering and does not include content-based or hybrid recommendation options. Future work could include integrating content-based filtering (CBF) components that use item metadata (e.g., genres, directors, actors, textual descriptions) and user profile attributes to better represent gray sheep users, especially when rating data is sparse or inconsistent. A hybrid neutrosophic-CF-CBF architecture can dynamically switch or mix collaborative and content-based signals based on each user’s uncertainty profile, potentially reducing recommendation variation and improving robustness for gray sheep users in real-world, multi-modal platforms.

The current study validated the static MovieLens 100 K and 1 M benchmark datasets; therefore, extending the evaluation to real-time interaction data and LLM-generated synthetic datasets has been identified as an important direction for future work to further assess robustness and generalization. Furthermore, future work will expand the validation protocol beyond static benchmark datasets by incorporating real-time user-item interaction data from modern recommendation platforms, allowing the proposed framework to be evaluated in the context of continuously evolving preference patterns and streaming scenarios. A complementary approach is to use synthetic user-item rating datasets generated by multiple large language models (LLMs) to systematically vary gray sheep prevalence, sparsity levels, and rating distributions, thereby providing a controlled setting for further testing the neutrosophic clustering-based approach’s robustness and generalization capability. When these techniques are combined, they form a road map for developing recommender systems that better represent and serve gray sheep customers while staying compatible with existing pipelines for mainstream users. Compared to NCF, LightGCN, and AutoRec, the suggested neutrosophic k-means clustering-IBCF architecture outperforms modern deep learning-based recommenders for gray-sheep users, despite their better representation learning capacity. This shows that systematic uncertainty modeling is a useful design axis for deep architectures and can be securely integrated into current neural pipelines. In order to enable adaptive user classification based on changing rating behaviors, the inquiry will also incorporate dynamic thresholding for the indeterminacy parameter ($\:I\left(x\right)$). Additionally, there are plans to evaluate scalability in a variety of domains other than movie recommendations and to extend the neutrosophic clustering approach to larger, sparser datasets.

## Data Availability

The datasets analyzed during the current study are publicly available as follows:- MovieLens 100 K: [https://grouplens.org/datasets/movielens/100k/](https:/grouplens.org/datasets/movielens/100k)- MovieLens 1 M: [https://grouplens.org/datasets/movielens/1m/](https:/grouplens.org/datasets/movielens/1m)- Book-Crossing: [https://www.kaggle.com/datasets/syedjaferk/book-crossing-dataset](https:/www.kaggle.com/datasets/syedjaferk/book-crossing-dataset)- Last.fm Dataset: [https://grouplens.org/datasets/hetrec-2011/](https:/grouplens.org/datasets/hetrec-2011)No new data were generated during this study.
